# Optimizing PV/T collector performance using coupled solid and porous layer configurations

**DOI:** 10.1038/s41598-026-51541-8

**Published:** 2026-05-21

**Authors:** S. Boulhidja, A. Bourouis, T. E. Boukelia, A. Tanougast, K. Hriczó

**Affiliations:** 1 Mechanical Engineering Department, Mouloud Mammeri University, Tizi-Ouzou, Algeria; 2 Mechanical Engineering Laboratory, Faculty of Science and Technology, Mohamed Seddik Benyahia University, Jijel, Algeria; 3 Mechanical and Advanced Materials Laboratory, Polytechnic School of Constantine, Constantine, Algeria; 4 Institute of Mathematics, Faculty of Mechanical Engineering and Informatics, University of Miskolc, Miskolc Egyetemvaros, Miskolc, 3515 Hungary

**Keywords:** Photovoltaic/thermal (PV/T) collector, Solid–porous coupled layer, Convective heat transfer, Numerical simulation, Thermal and electrical efficiency enhancement, Energy science and technology, Engineering, Materials science

## Abstract

Recent research on photovoltaic/thermal (PV/T) collectors has focused on two key strategies to enhance performance: geometric modifications of the thermal flow channel (such as fins, baffles, and ribbed structures) and the integration of advanced materials like phase change materials (PCMs) and porous media to improve heat transfer and overall efficiency. In this direction, this numerical study investigates the performance enhancement of a photovoltaic/thermal (PV/T) solar collector through the integration of a solid layer along the lower wall of the airflow channel, coupled with a porous medium. The solid layer is introduced to accelerate the airflow and intensify convective heat transfer, thereby improving the thermal management of the photovoltaic cells. To solve the governing transport equations, an in-house computational code was developed in the Fortran programming language based on the finite volume method, coupled with the SIMPLER algorithm. The effects of solid-layer thickness and length, porous-layer thickness, and Darcy number are systematically investigated under a constant Reynolds number (Re = 500) and a uniform heat flux of 1000 W/m². The obtained results show that increasing the solid-layer thickness significantly enhances airflow acceleration and leads to a pronounced reduction in PV cell temperature of up to 33 °C. Extending the solid-layer length further improves the convective cooling process and increases both electrical and thermal efficiencies. When combined with a sufficiently permeable porous layer, additional performance gains are achieved, particularly at high Darcy numbers (Da = 10^− 1^). Compared to a conventional PV/T collector, the optimized configuration demonstrates enhancements of up to 60% in thermal efficiency and 28% in electrical efficiency.

## Introduction

Fossil fuels have long powered global energy systems, but their use incurs significant environmental consequences, including the acceleration of climate change. Moreover, their rising costs and finite availability underscore the urgent need to shift toward sustainable and environmentally friendly energy alternatives, such as renewable resources. Among them, solar power stands out as a promising pathway and it using for different techniques including thermal collectors of various types, Trombe wall, Photovoltaic panels, …, ect. Globally, photovoltaic (PV) technology is widely deployed, with PV panels typically converting solar irradiation into electricity at efficiencies ranging from 12% to 22%^1^. However, photovoltaic performance is strongly temperature-dependent, with higher cell temperatures leading to reduced electrical efficiency and accelerated material degradation. Consequently, significant research is now dedicated to optimizing PV performance through effective cooling solutions. One notable approach is the integration of PV modules with thermal collectors to form hybrid photovoltaic–thermal (PV/T) systems. In such systems, cooling the PV panels not only enhances electrical efficiency but also enables the simultaneous production of electricity and useful thermal energy.

Recently, photovoltaic/thermal (PV/T) systems have attracted significant research attention, with numerous studies focused on their development and optimization to enhance both performance and overall efficiency. Among the various enhancement strategies, geometric modifications of the thermal flow channel are particularly attractive, as they can yield significant performance improvements while imposing minimal additional cost and system complexity. Such enhancements can be achieved through several design approaches, including modifications to the absorber plate by integrating features such as wavy fins, discontinuous fins, baffles, corrugated absorber plates, and ribbed structures. In this category, Jha et al.^2^ carried out an experimental energy and exergy comparison of photovoltaic–thermal air collectors using a flat plate absorber (Model-I) and a novel wavy plate absorber (Model-II). The results demonstrated that Model-II significantly outperformed Model-I, achieving 16% higher thermal energy, 27.4% higher thermal exergy, and a 1.2% increase in net electrical output. Özakin et al.^3^ conducted an experimental and numerical study on the energy and exergy performance of an air-based photovoltaic/thermal (PV/T) system with different fin configurations. Using ANSYS Fluent, panel surface temperature and airflow characteristics were analyzed and validated against experimental data. Compared with an empty channel, sparse and frequent fins enhanced thermal efficiency by approximately 55% and 70%, respectively, while reducing PV panel surface temperature by 10–15 °C. Tonui et al.^4^ designed and compared two innovative systems to improve the cooling process of hybrid PV/T solar collectors. The first system (TMS) incorporated a flat plate in the middle of the channel, while the other system (FIN) used fins attached to the rear wall of the channel. The results indicated that the finned system (FIN) achieved a thermal efficiency of 43%, followed by the flat plate system (TMS) at 35%, whereas the reference system recorded an efficiency of 30%. Aspetakis et al.^5^ investigated heat transfer enhancement techniques for air-based photovoltaic/thermal (PV/T) systems. A novel V-baffle configuration was developed and experimentally tested to validate a CFD model. The V-baffle configuration shows an average reduction in PV cell temperature of approximately 8 °C, achieving up to 22% higher thermal enhancement rates compared to conventional designs. An experimental investigation on a naturally ventilated double-skin façade integrating semi-transparent PV modules in a nearly zero-energy building was conducted by Gür et al.^6^. The system featured an 18 cm air cavity with operable vents, yielding winter heating gains ranging from 70 to 135 °C·h. The results also revealed strong orientation-dependent effects, with peak performance occurring in the east during the morning, south at midday, and west in the afternoon. On an annual basis, the system generated 2.43 MWh of electricity, avoided 1.457 tCO₂ emissions, and provided an economic benefit of approximately USD 114.

On the other hand, a substantial body of research has focused on improving the properties of coolant fluids and evaluating their impact on the overall efficiency of PV/T systems. In this context, Kahani et al.^7^ conducted both experimental and analytical investigations to evaluate the enhancement of PV module performance using a water/titanium dioxide (TiO₂) nanofluid circulated through a semicircular cooling pipe beneath the PV module. Their findings revealed that using a nanofluid with 0.5% mass concentration, rather than conventional water, enhanced the system’s thermal efficiency by about 27%. Jidhesh et al.^8^ examined a semitransparent PV/T system through analytical and experimental methods, employing CuO nanofluid as the coolant. The results showed that conventional water lowered the panel temperature by roughly 9 °C, whereas CuO nanofluid achieved a reduction of about 12 °C, demonstrating its enhanced cooling performance.

Furthermore, integrating phase change materials (PCMs) can significantly enhance the performance of PV solar collectors. By storing thermal energy over extended periods, these materials help maintain lower temperatures for the photovoltaic panels^9^. Consequently, numerous studies in the literature have explored this approach. Choubineh et al.^10^ conducted an experimental investigation to improve the thermal management of a PV/T air collector by integrating a 6 mm layer of PCM (salt-hydrate) on the rear surface of the PV module. Their findings showed that the modified configuration decreased the PV surface temperature by approximately 3.7–4.3 °C. Maarof et al.^11^ investigated a double-pass PVT system integrating phase change materials and wavy fins (PVT-PCMWF) to enhance heat transfer. Their CFD results showed an increase in outlet air temperature from 41 °C to 44 °C (+ 7.75%), accompanied by a significant improvement in thermal efficiency from 11.15% to 18% (+ 61.35%) and a 2% rise in electrical efficiency (up to 12.09%). In addition, the daily heat gain increased by 72% (229.6 W compared to 133.5 W). In another investigation, Hasan et al.^12^ integrated a PCM layer behind a photovoltaic panel to evaluate its impact on thermal management. The results indicated that the addition of the PCM significantly reduced the front and back surface temperatures of the PV panel by 12.3 °C and 22.6 °C, respectively. This improved thermal regulation translated into higher electrical output, with peak and average power increasing by 7.2% and 5.5%, respectively. A hybrid cooling approach in a PVT system, combining ZnO/water nanoparticles with paraffin-based PCM, was implemented by Sardarabadi et al.^13^. They investigated two configurations: one incorporating both nanoparticles and PCM, and a reference model using only water. Their results menstruated that the combined use of PCM and nanoparticles increased electrical efficiency by 13% compared to the reference system. Maarof et al.^14^ proposed a bi-fluid PV/T system integrating finned tubular heat exchangers with phase change materials (Bi-fluid PV/T-PCM-TFT) to improve thermal management. Their CFD simulations showed a PV temperature reduction of 8.7 °C (41.3 °C compared to 49.4 °C), leading to a 4% increase in electrical efficiency (12.94% compared to 12.44%) and a 4.44% enhancement in daily energy output. Furthermore, increasing the mass flow rate from 0.01 to 0.03 kg/s improved overall system performance from 58.1% to 74%. Gür et al. 15 investigated a solar-assisted domestic radiator for nearly zero-energy buildings using experimental and numerical methods. The PV/T-powered system employed nano-enhanced phase change materials (NEPCM) with B₄C nanoparticles to improve heat transfer. Results showed enhanced indoor temperature regulation, with a maximum difference of 2.82 K compared to systems without PCM. NEPCM also provided ~ 35% higher thermal retention. Additionally, heat storage capacity increased by 20% relative to conventional PCM. In another study, Gür et al.^16^ investigated a solar-assisted underfloor heating system for nearly zero-energy buildings using a PV/T collector and nano-enhanced PCM with 1% Cu nanoparticles. Numerical simulations under turbulent flow, performed via the finite volume method, revealed that pure PCM provided the greatest thermal benefit, with a maximum temperature rise of 4 K compared to systems without PCM. The inclusion of nanoparticles yielded only a marginal improvement of 0.1 K. Maarof et al.^17^ investigated a PVT system combining phase change materials (PCM) with single-finned tube cooling for enhanced thermal regulation. Their CFD results showed a significant reduction in PV temperature from 75.3 °C (conventional PV) to 45.8 °C, leading to an increase in electrical efficiency up to 13%, compared to 11.6% for conventional PV, 12.3% for PV-PCM, and 12.8% for standard PV/T systems. Furthermore, increasing the water flow rate from 0.02 to 0.06 kg/s improved electrical and thermal efficiencies by 0.92% and 23.6%, respectively. Many more works have been reported in the literature to investigate the use of phase change materials (PCMs) to improve the thermal and electrical performance of PVT collectors^18–21^.

On the other hand, the incorporation of porous materials is regarded as one of the most effective techniques for enhancing the performance of heat transfer systems, as it increases both thermal conductivity and the heat transfer surface area. This approach has been extensively investigated in the thermal engineering literature and has proven highly effective in improving heat transfer across diverse applications^22,23^. Indeed, numerous studies have specifically examined the role of porous materials in enhancing PV/T system performance. In this framework, Asefi et al.^24^ carried out a numerical investigation on the integration of porous media—specifically copper foam, aluminum foam, and expanded graphite—within PCM-enhanced PV/T systems. Their findings demonstrated that coupling PCM with porous structures markedly enhances electrical, thermal, and exergy efficiencies. Among the tested materials, copper foam exhibited the highest performance, largely attributable to its superior thermal conductivity. Using ANSYS Fluent, Tahmasbi et al.^25^ numerically investigated the influence of a porous medium in a two-dimensional PV/T system, focusing on key parameters such as the Reynolds number, porous layer thickness, and incident solar flux. The results showed that incorporating porous media substantially enhanced conductive heat transfer, reducing PV cell temperatures by 25–40 °C and increasing electrical efficiency by about 3%. At the same time, the outlet fluid temperature rose by 10–17 °C, leading to a 10–40% improvement in thermal efficiency. Essa et al.^26^ experimentally compared two water-cooled PV/T configurations: one incorporating a phase change material (PCM) and another combining PCM with porous metallic media (PMM). Paraffin wax and stainless steel were used as the PCM and PMM, respectively. The results indicated that for all investigated flow rates, the PV/T system incorporating PCM–PMM exhibited superior electrical and thermal performance compared with the PCM-only system. Sangtarash et al.^27^ conducted three-dimensional numerical simulations to examine the integration of porous media in PV/T systems, considering three spatial configurations (top-filled, middle-filled, and fully filled) and various materials, including metal and ceramic foams. Their results showed that the fully filled configuration achieved the highest thermal efficiency enhancement (32%), compared with 24% and 17% for the top- and middle-filled designs, respectively. Using MATLAB simulations, Mustafa et al.^28^ investigated the influence of the positioning of ∇-shaped porous metal foam on the performance of a PV/T system. The porous foam was installed at three different locations within the fluid channels: beneath the photovoltaic panels, above the insulated surface, and simultaneously at both locations. The results showed that the maximum overall energy efficiency ranged from 61.53% to 111.49%, while the overall exergetic efficiency varied between 14.18% and 17.46%, compared with the PV/T system without porous metal foam. Özşimşek et al.^29^ numerically investigated the thermal performance of a PV/T system equipped with water-based cooling tubes containing a porous medium. Their results showed that, at a low mass flow rate of 0.3 L/min, the inclusion of the porous medium significantly enhanced heat transfer, leading to an increase in thermal efficiency of up to 118%.

Moreover, the literature review reveals that the performance of photovoltaic/thermal (PV/T) systems can be significantly enhanced through modifications to the cooling channel geometry, such as the incorporation of solid layers or baffles^3,5^, as well as through the integration of cooling enhancement techniques, including phase change materials (PCMs), nanofluids, and porous media^8,16,28^, thereby improving thermal management.

However, despite these advances, and to the best of the authors’ knowledge, the combined use of a solid layer and a porous medium within the airflow channel of PV/T systems has not yet been investigated. Specifically, the coupled effects of enhanced conductive heat transfer within the porous layer and improved convective heat transfer resulting from flow acceleration due to the reduced passage area imposed by the solid layer remain unexplored in the context of PV/T cooling performance. This gap is noteworthy, as such a hybrid configuration may provide a synergistic mechanism for improving heat extraction, temperature uniformity, and overall system efficiency. Accordingly, this study introduces a novel PV/T design that integrates both solid and porous structures, aiming to achieve superior thermal management through a relatively simple and effective configuration. It should be noted that the present investigation builds upon a recent study^30^, which examined mixed convection in an air-cooled PV/T solar collector incorporating a porous medium. Building on this foundation, the current work systematically explores the influence of solid-layer thickness and length, porous-layer thickness, and Darcy number on the thermal and electrical performance of a PV/T collector.

## Data and methodology

### Physical models

**Fig. 1:**
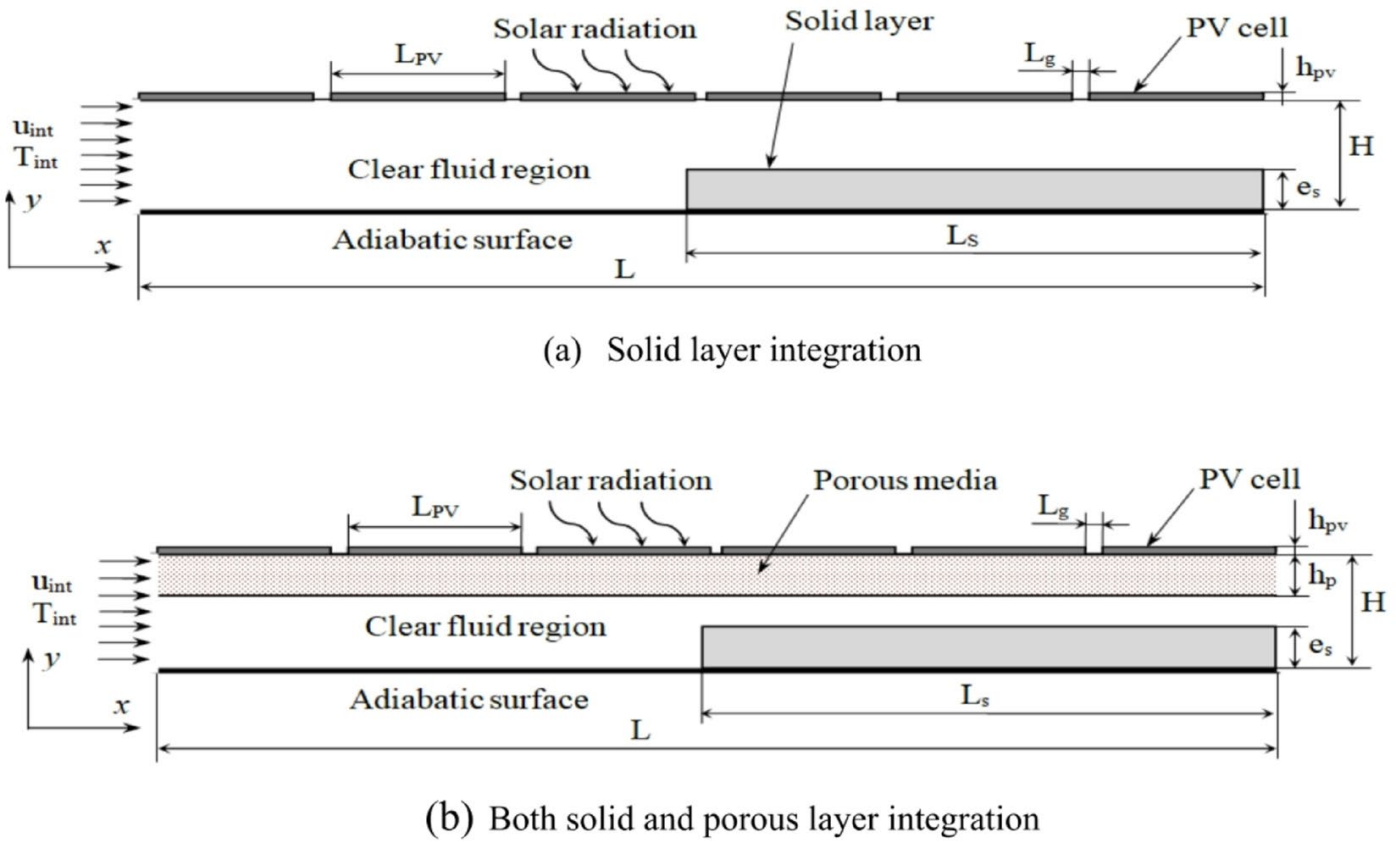
2D schematic representation of the PVT collector.

As indicated previously, this study presents a numerical investigation of an air-based photovoltaic–thermal (PV/T) solar system. The system consists of a coverless photovoltaic panel directly coupled to an air flow channel, as illustrated in Fig. 1. Two distinct configurations are examined. In the first configuration (Fig. 1.a), a solid polypropylene layer is installed along the bottom wall of the air channel. In the second configuration (Fig. 1.b), a porous metallic foam layer is attached to the underside of the PV cells, while retaining the solid polypropylene layer.

The different specifications of the investigated PV/T system, including its geometric dimensions are presented in Table 1. In addition, Table 2 summarizes the main thermo-physical properties of the materials used in the studied PV/T solar collector with its two layouts.

**Table 1:** Technical specifications of the investigated photovoltaic–thermal (PVT) panel.

	PV cell length	$\:{\mathrm{L}}_{\mathrm{PV}}=180\:\mathrm{m}\mathrm{m}$
	Spacing between cells	Lg $\:=$24 mm
Photovoltaic panel (PV)	PV cell height	$\:{\mathrm{h}}_{\mathrm{pv}}=\:$4 mm
	Reference efficiency	$\:{\eta}_{\mathrm{ref}}=$15%^31^
	Silicon efficiency temperature coefficient	$\:\upbeta _{0} = \:$0.0045 $\:{\mathrm{k}}^{\mathrm{-1}}$^31^
Fluid channel	Length	L $\:=$1200 mm
	Height	H $\:=$ 48 mm
Aluminium porous layer	Dimensionless thickness ($\:\mathrm{Ep}\mathrm{=}{\mathrm{h}}_{\mathrm{P}}/\mathrm{H})$	Ranging (0–0.7)
Polypropylene solid layer	Length	$\:{\mathrm{L}}_{\mathrm{S}}=\:$0.25 L, 0.5 L and 0.75 L
	Dimensionless thickness ($\:\mathrm{E}\mathrm{s}\mathrm{=}{\mathrm{e}}_{\mathrm{s}}/\mathrm{H})$	Ranging (0–0.7)

**Table 2:** Thermo-physical properties of the investigated photovoltaic–thermal (PVT) panel^25^.

Properties	$\:\uprho \:({\mathrm{kg}}\:{\mathrm{m}}^{{ - 3}} )$	$\:{\mathrm{C}}_{\mathrm{P}}\mathrm{\:(J}\text{}{\mathrm{kg}}^{\mathrm{-1}}\:{\mathrm{K}}^{\mathrm{-1}}\mathrm{)}$	$\:\mathrm{K\:(W}\text{}{\mathrm{m}}^{\mathrm{-1}}\:{\mathrm{K}}^{\mathrm{-1}}\mathrm{)}$	$\:\upmu \times 10^{{ - 5}} ({\mathrm{kg}}\:{\mathrm{m}}^{{ - 1}} \:{\mathrm{s}}^{{ - 1}} )$
PV Cell	2330	700	148.4	–
Aluminium	2179	871	202	–
Polypropylene	960	2000	0.3	–
Air	1.125	1006.4	0.0242	1.78

**Table 3:** Effective properties of the porous medium (Aluminum + air) for $\:\epsilon\:=0.98.$

Properties	$\:\rho _{{{\mathrm{eff}}}} {\mathrm{(kg}}\;{\mathrm{m}}^{{ - {\mathrm{3}}}} {\mathrm{)}}$	$\:{\mathrm{Cp}}_{{{\mathrm{eff}}}} {\mathrm{(J}}\,{\mathrm{kg}}^{{ - {\mathrm{1}}}} \,{\mathrm{K}}^{{ - {\mathrm{1}}}} {\mathrm{)}}$	$\:{\mathrm{k}}_{{{\mathrm{eff}}}} {\mathrm{(W}}\,{\mathrm{m}}^{{ - {\mathrm{1}}}} \,{\mathrm{K}}^{{ - {\mathrm{1}}}} {\mathrm{)}}$	$\:\upmu _{{{\mathrm{eff}}}} \times 10^{{ - 5}} ({\mathrm{kg}}\,{\mathrm{m}}^{{ - 1}} \,{\mathrm{s}}^{{ - 1}} )$
Value	44.7	873	4.06	1.816

The effective properties of the porous medium are determined using the following analytical expressions^32–34^.

1 $$\:{\boldsymbol{\rho\:}}_{\mathrm{e}\mathrm{f}\mathrm{f}}={\upepsilon\:}{\boldsymbol{\rho\:}}_{\mathrm{f}}+\left(1-{\upepsilon\:}\right){\boldsymbol{\rho\:}}_{\mathrm{s}}$$

2 $$\:{\mathrm{C}\mathrm{p}}_{\mathrm{e}\mathrm{f}\mathrm{f}}=\frac{{\upepsilon\:}{{\boldsymbol{\rho\:}}_{\mathrm{f}}\mathrm{C}\mathrm{p}}_{\mathrm{f}}+(1-{\upepsilon\:}){\boldsymbol{\rho\:}}_{s}{\mathrm{C}\mathrm{p}}_{\mathrm{s}}}{{\boldsymbol{\rho\:}}_{\mathrm{e}\mathrm{f}\mathrm{f}}}$$

3 $$\:{\mathrm{k}}_{\mathrm{e}\mathrm{f}\mathrm{f}}={\upepsilon\:}{\mathrm{k}}_{\mathrm{f}}+(1-{\upepsilon\:}){\mathrm{k}}_{\mathrm{s}}$$

4 $$\:\upmu _{{{\mathrm{eff}}}} = \frac{{\upmu _{{\mathrm{f}}} }}{\upvarepsilon }$$

In Table 3, $\:{\rho\:}_{\mathrm{f}}{,\:\mathrm{C}\mathrm{p}}_{\mathrm{f}}$ and $\:{\mathrm{k}}_{\mathrm{f}}$ represent the thermophysical properties of air, while $\:{\rho\:}_{\mathrm{s}}{,\:\mathrm{C}\mathrm{p}}_{\mathrm{s}}$ and $\:{\mathrm{k}}_{\mathrm{s}}$ correspond to the thermophysical properties of aluminum listed in Table 2.

### Governing equations

For the purpose of modeling the physical phenomena incorporated in the investigated system with its two layouts, the following assumptions are adopted:

- The solar radiation is considered constant and perpendicular to the photovoltaic panel.
- The porous medium is assumed to be homogeneous and isotropic.
- The local thermal equilibrium (LTE) assumption is considered between the two phases of the porous medium.
- The thermo-physical properties are assumed to be uniform and constant.
- The lower surface of the canal is considered adiabatic (insulated).
- The fluid flow is assumed to be laminar and incompressible, and the problem is simulated using a two-dimensional approach^35^.

Under the above assumptions, and by using the dimensionless variables defined below in Eq. (5), the governing equations describing forced convection throughout the entire channel can be expressed in dimensionless form as follows^25,36^:

5 $$\:\left(\mathrm{X},\mathrm{Y},{\mathrm{E}}_{\mathrm{P}},\:{\mathrm{E}}_{\mathrm{S}}\right)=(\mathrm{x},\mathrm{y},{\mathrm{h}}_{\mathrm{P}},{\mathrm{e}}_{\mathrm{S}})/\mathrm{H},\:\left(\mathrm{U},\mathrm{V}\right)=(\mathrm{u},\mathrm{v})/{\mathrm{U}}_{\mathrm{i}\mathrm{n}\mathrm{t}},\:{\uptheta\:}={\mathrm{k}}_{\mathrm{f}}(\mathrm{T}-{\mathrm{T}}_{\mathrm{i}\mathrm{n}\mathrm{t}})/{\mathrm{Q}}_{\mathrm{U}}\mathrm{H},\:\mathrm{P}=\mathrm{p}/{{\uprho\:}}_{\mathrm{f}}{\mathrm{U}}_{\mathrm{i}\mathrm{n}\mathrm{t}}^{2}$$

#### Continuity equation

6 $$\:\mathrm{U}\frac{\partial\:\mathrm{U}}{\partial\:\mathrm{X}}+\mathrm{V}\frac{\partial\:\mathrm{U}}{\partial\:\mathrm{Y}}=0$$

#### Momentum equation

7 $$\:\frac{1}{{{\upepsilon\:}}^{2}}\left(\mathrm{U}\frac{\partial\:\mathrm{U}}{\partial\:\mathrm{X}}+\mathrm{V}\frac{\partial\:\mathrm{U}}{\partial\:\mathrm{Y}}\right)=-\frac{\partial\:\mathrm{P}}{\partial\:\mathrm{X}}+\frac{{\mathrm{R}}_{{\upmu\:}}}{{\mathrm{R}}_{\mathrm{e}}}\left(\frac{{\partial\:}^{2}\mathrm{U}}{\partial\:{\mathrm{X}}^{2}}+\frac{{\partial\:}^{2}\mathrm{U}}{\partial\:{\mathrm{Y}}^{2}}\right)-{\uplambda\:}(\frac{1}{{\mathrm{R}}_{\mathrm{e}}{\mathrm{D}}_{\mathrm{a}}}\mathrm{U}+\mathrm{F}\mathrm{U}\sqrt{{\mathrm{U}}^{2}+{\mathrm{V}}^{2}})$$

8 $$\:\frac{1}{{{\upepsilon\:}}^{2}}\left(\mathrm{U}\frac{\partial\:\mathrm{V}}{\partial\:\mathrm{X}}+\mathrm{V}\frac{\partial\:\mathrm{V}}{\partial\:\mathrm{Y}}\right)=-\frac{\partial\:\mathrm{P}}{\partial\:\mathrm{X}}+\frac{{\mathrm{R}}_{{\upmu\:}}}{{\mathrm{R}}_{\mathrm{e}}}\left(\frac{{\partial\:}^{2}\mathrm{V}}{\partial\:{\mathrm{X}}^{2}}+\frac{{\partial\:}^{2}\mathrm{V}}{\partial\:{\mathrm{Y}}^{2}}\right)-{\uplambda\:}(\frac{1}{{\mathrm{R}}_{\mathrm{e}}{\mathrm{D}}_{\mathrm{a}}}\mathrm{V}+\mathrm{F}\mathrm{U}\sqrt{{\mathrm{U}}^{2}+{\mathrm{V}}^{2}})$$

*Energy equation for the channel*

9 $$\:\mathrm{U}\frac{\partial\:{\uptheta\:}}{\partial\:\mathrm{X}}+\mathrm{V}\frac{\partial\:{\uptheta\:}}{\partial\:\mathrm{Y}}=\frac{{\mathrm{K}}_{\mathrm{r}}}{{\mathrm{P}}_{\mathrm{r}}{\mathrm{R}}_{\mathrm{e}}}\left(\frac{{\partial\:}^{2}{\uptheta\:}}{\partial\:{\mathrm{X}}^{2}}+\frac{{\partial\:}^{2}{\uptheta\:}}{\partial\:{\mathrm{Y}}^{2}}\right)$$

*Energy equation for the PV cells and solid layer*:

10 $$\:0=\frac{{\partial\:}^{2}{\uptheta\:}}{\partial\:{\mathrm{X}}^{2}}+\frac{{\partial\:}^{2}{\uptheta\:}}{\partial\:{\mathrm{Y}}^{2}}$$

In the previous equations: $$\:\left\{\begin{array}{c}{\:\mathrm{k}}_{\mathrm{r}}=1,\:\:\lambda\:=0\:,\:\:\epsilon\:=1\:\:\:\:\:\:\:\:\:\:\:\:\:\:\:\:\:\:\:\:\:\:\:\:\:in\:the\:clear\:fluid\:\:\:\:\:\:\\\:{{\:\:\mathrm{k}}_{\mathrm{r}}=\:\mathrm{k}}_{\mathrm{e}\mathrm{f}\mathrm{f}}/{\:\mathrm{k}}_{\mathrm{f}},\:\:\lambda\:=1,\:\:\epsilon\:=0.98\:\:\:\:\:\:\:in\:the\:porous\:media\end{array}\right.$$

Where:

$$\:\:\mathrm{R}\mathrm{e}=\frac{\mathrm{H}{\mathrm{U}}_{\mathrm{i}\mathrm{n}\mathrm{t}}}{{\upupsilon\:}}\:,\:Da=\frac{\mathrm{K}}{{\mathrm{H}}^{2}}\:,\:\:Pr=\frac{{\upmu\:}{\mathrm{C}}_{\mathrm{P}}}{{\mathrm{k}}_{\mathrm{f}}}\:,\:\mathrm{F}=\frac{{\mathrm{C}}_{\mathrm{F}}\mathrm{H}}{\sqrt{\mathrm{K}}}\:,\:{\Delta\:}\mathrm{T}=\frac{{\mathrm{Q}}_{\mathrm{U}}\mathrm{H}}{{\mathrm{k}}_{\mathrm{f}}},\:{\mathrm{K}}_{\mathrm{r}}=\frac{{\mathrm{k}}_{\mathrm{e}\mathrm{f}\mathrm{f}}}{{\mathrm{k}}_{\mathrm{f}}}\:,\:{\mathrm{R}}_{{\upmu\:}}=\frac{{{\upmu\:}}_{\mathrm{e}\mathrm{f}\mathrm{f}}}{{{\upmu\:}}_{\mathrm{f}}}$$

The values of the permeability can be calculated by the Kozeny-Carman formula as^25^:

11 $$\:\mathrm{K}=\frac{{d}_{p}^{2}\epsilon\:}{150{\left(1-\epsilon\:\right)}^{2}}$$

Where: $\:{d}_{p\:}$is the pore diameter of the porous media.

Furthermore, the corresponding dimensionless boundary conditions of this problem can be summarized in Table 4.

Moreover, the performance of the system is assessed using both thermal and electrical efficiencies formulations. The thermal efficiency is expressed as follows^37^:

12 $$\:{{\upeta\:}}_{\mathrm{T}\mathrm{h}}=\frac{{\mathrm{Q}}_{\mathrm{u}}}{\mathrm{I}{\mathrm{A}}_{\mathrm{c}}}$$

Where $\:{\mathrm{Q}}_{\mathrm{u}}$ represents the amount heat transferred to the coolant, and can be expressed as follows^37^:

13 $$\:{\mathrm{Q}}_{\mathrm{u}}=\dot{\mathrm{m}}\times\:{\mathrm{C}}_{\mathrm{P}}\times\:({\mathrm{T}}_{\mathrm{o}\mathrm{u}\mathrm{t}\mathrm{l}\mathrm{e}\mathrm{t}}-{\mathrm{T}}_{\mathrm{i}\mathrm{n}\mathrm{t}})$$

In this expression, $\:{\:\mathrm{C}}_{\mathrm{P}}$, is the specific heat capacity of the air, while $\:{\mathrm{T}}_{\mathrm{i}\mathrm{n}\mathrm{t}}$ and $\:{\mathrm{T}}_{\mathrm{o}\mathrm{u}\mathrm{t}\mathrm{l}\mathrm{e}\mathrm{t}}$ denote its average inlet and outlet temperatures, respectively. The mass flow rate ($\:\dot{\mathrm{m}}$) of the working fluid can be determined from the following relation:

14 $$\:\dot{\mathrm{m}}={\uprho\:}\times\:\mathrm{H}\times\:{\mathrm{U}}_{\mathrm{i}\mathrm{n}\mathrm{t}}$$

With the inlet velocity ($\:{\mathrm{U}}_{\mathrm{i}\mathrm{n}\mathrm{t}}$) depends on the Reynolds number value.

Moreover, the average outlet temperature in this case can be estimated using the following formulation^33^:

15 $$\:{\mathrm{T}}_{\mathrm{O}\mathrm{u}\mathrm{t}\mathrm{l}\mathrm{e}\mathrm{t}}=\underset{0}{\overset{\mathrm{H}}{\int\:}}\mathrm{T}\mathrm{u}\mathrm{d}\mathrm{y}/\underset{0}{\overset{\mathrm{H}}{\int\:}}\mathrm{u}\mathrm{d}\mathrm{y}$$

**Table 4:** Thermal and dynamic dimensionless boundary conditions.

Bottom wall	U = V=0, $\:\frac{{\partial \uptheta }}{{\partial {\mathrm{Y}}}} = {\mathrm{0}}$
Top wall	U = V=0, $\:\frac{{\partial \uptheta }}{{\partial {\mathrm{Y}}}} = \frac{{ - {\mathrm{1}}}}{{{\mathrm{Rk}}}}$
Inlet	U = 1, V = 0, $\:\uptheta = 0$
Outlet	$\:\frac{{\partial {\mathrm{U}}}}{{\partial {\mathrm{X}}}} = {\mathrm{0}},\:{\mathrm{V}} = {\mathrm{0}},\:\frac{{\partial \uptheta }}{{\partial {\mathrm{X}}}} = {\mathrm{0}}$
PV Cells/porous interface	$\:{\mathrm{U}} = {\mathrm{V}} = 0,\:\uptheta _{{\mathrm{c}}} {{ = }}\uptheta _{{\mathrm{P}}} ,\:\left( {\frac{{\partial \theta }}{{\partial {\mathrm{Y}}}}} \right)_{{\mathrm{c}}} = \frac{{{\mathrm{k}}_{{{\mathrm{eff}}}} }}{{{\mathrm{k}}_{{\mathrm{c}}} }}\left( {\frac{{\partial \uptheta }}{{\partial {\mathrm{Y}}}}} \right)_{{\mathrm{p}}}$
Porous/air interface	$\:{\mathrm{U}}_{{\mathrm{f}}} = {\mathrm{U}}_{{\mathrm{P}}} ,\:{\mathrm{V}}_{{\mathrm{f}}} = {\mathrm{V}}_{{\mathrm{P}}} ,\:\uptheta _{{\mathrm{f}}} = \uptheta _{{\mathrm{P}}} ,\:\left( {\frac{{\partial {\mathrm{U}}}}{{\partial {\mathrm{Y}}}} + \frac{{\partial {\mathrm{V}}}}{{\partial {\mathrm{Y}}}}} \right)_{{\mathrm{f}}} = {\mathrm{R}}_{\upmu } \left( {\frac{{\partial {\mathrm{U}}}}{{\partial {\mathrm{Y}}}} + \frac{{\partial {\mathrm{V}}}}{{\partial {\mathrm{Y}}}}} \right)_{{\mathrm{p}}} ,\:\left( {\frac{{\partial \uptheta }}{{\partial {\mathrm{Y}}}}} \right)_{{\mathrm{f}}} = {\mathrm{K}}_{{\mathrm{r}}} \left( {\frac{{\partial \uptheta }}{{\partial {\mathrm{Y}}}}} \right)_{{\mathrm{p}}}$
Polypropylene/air interface	U = V=0, $\:\uptheta _{{\mathrm{s}}} = \uptheta _{{\mathrm{f}}}$, $\:\left( {\frac{{\partial \uptheta }}{{\partial {\mathrm{Y}}}}} \right)_{{\mathrm{s}}} = \frac{{{\mathrm{k}}_{{\mathrm{f}}} }}{{{\mathrm{k}}_{{\mathrm{s}}} }}\left( {\frac{{\partial \uptheta }}{{\partial {\mathrm{Y}}}}} \right)_{{\mathrm{f}}}$

On the other hand, the electrical efficiency of the PV panel can be written as^38^:

16 $$\:{{\upeta\:}}_{\mathrm{E}\mathrm{l}}={{\upeta\:}}_{\mathrm{r}\mathrm{e}\mathrm{f}}(1-{{\upbeta\:}}_{0}\left({\mathrm{T}}_{\mathrm{P}\mathrm{V}}-298\right))$$

Where $\:{{\upeta\:}}_{\mathrm{r}\mathrm{e}\mathrm{f}}$ represents the electrical efficiency of the photovoltaic panel under reference operating conditions, and $\:{{\upbeta\:}}_{0}$ denotes the power–temperature coefficient of the PV module (both parameters are given in Table 1), and $\:{\mathrm{T}}_{\mathrm{P}\mathrm{V}}$is the average temperature of the photovoltaic cells.

The fan power necessary to ensure air circulation is estimated based on the following expression^39,40^

17 $$\:{F}_{p}=\frac{{\dot{m}}_{air}{\Delta\:}P}{{{\rho\:}_{air}\eta\:}_{Fan}{\eta\:}_{Motor}}$$

Where $\:{\eta\:}_{Fan}$​ and $\:{\:\eta\:}_{Motor}$denote the fan and motor efficiencies, respectively, taken as 0.7 and 0.9^41^.

## Numerical analysis and code validation

A FORTRAN code was developed based on the finite volume method and the SIMPLER algorithm to solve the momentum, energy, and continuity equations. The power-law differencing scheme is employed to discretize the convective terms in the finite volume equations, while the central differencing scheme is used for the diffusion terms. The discretized governing equations for momentum and energy are solved using the Tri-Diagonal Matrix Algorithm (TDMA). The iterative procedure is carried out until the relative convergence criteria for all variables (U, V, and T) are satisfied with a tolerance lower than 10^− 6^.

**Fig. 2:**
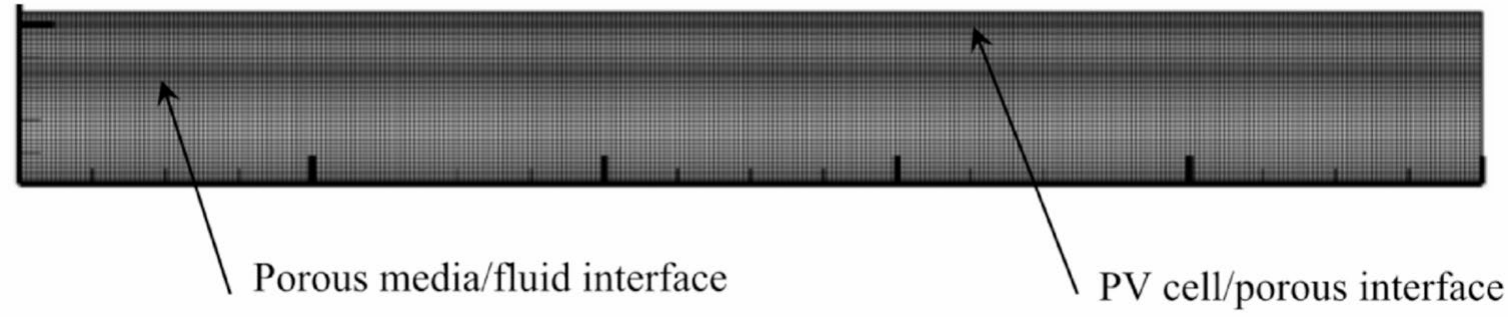
The structured mesh of the system at Ep = 0.3.

**Fig. 3:**
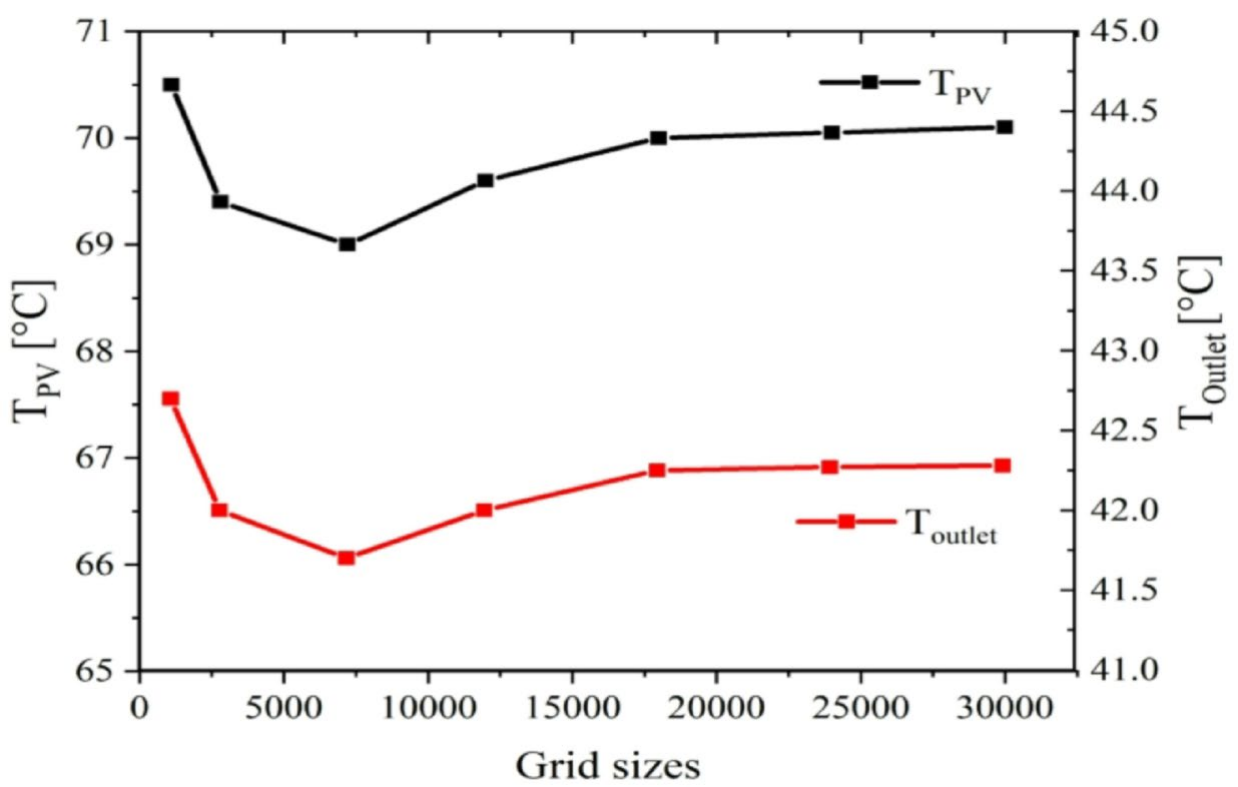
Grid sizes effect on the average outlet fluid and PV cell temperatures for Es = 0.5, Ls = 0.5 L, Re = 500.

A mesh independence study was conducted using a series of non-uniform grids consisting of 1100, 2800, 7200, 12,000, 18,000, 24,000 and 30,000 control volumes (Fig. 2), in order to ensure that the numerical predictions were independent of the grid resolution. As illustrated in Fig. 3, the variations in the fluid outlet temperature and the PV cell temperature progressively diminish with increasing grid density and become negligible for meshes exceeding 18,000 nodes. The maximum deviation between the 18,000 and 30,000 node grids was less than 0.1%, confirming that grid-independent solutions are achieved beyond this threshold.

Based on these observations, a mesh of 18,000 nodes (60 × 300) was adopted for all simulations, as it provides an optimal balance between computational accuracy and efficiency while ensuring reliable prediction of the thermal and flow fields.

**Fig. 4:**
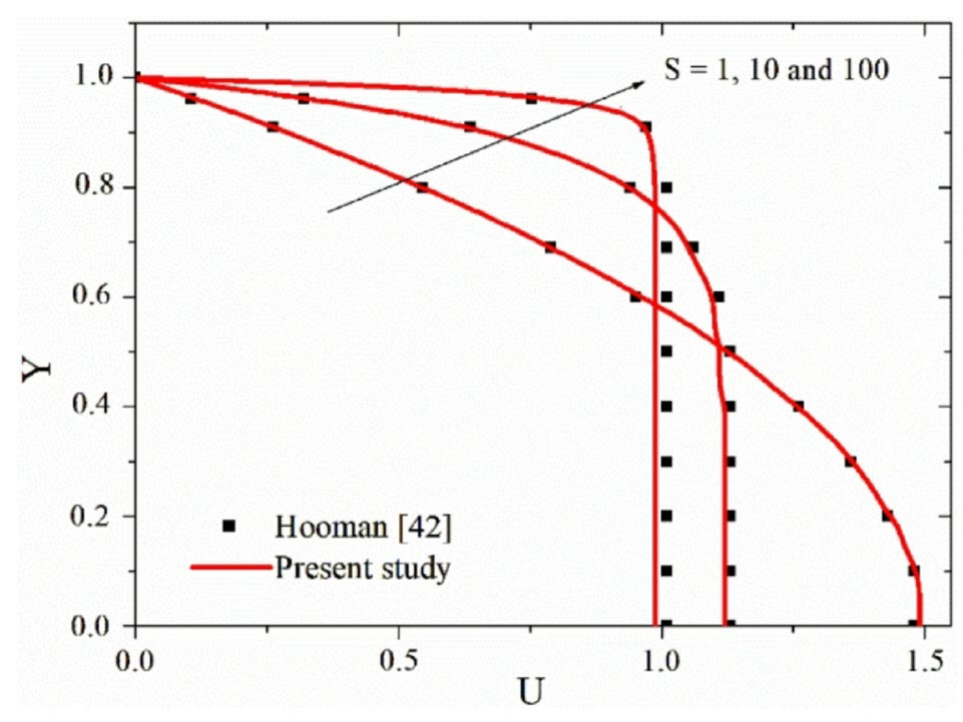
Validation of the U-velocity profiles with those of Hooman^42^, as a function of S (S =$\:1/\sqrt{Da}$).

To assess the accuracy of our numerical results, two validation tests were performed. The first validation involved a comparison with an analytical solution reported by Hooman^42^ for forced convection in a horizontal porous duct. As illustrated in Fig. 4, an excellent agreement was observed between the dimensionless U-velocity profiles predicted in this study and the analytical results across various Darcy numbers.

**Fig. 5:**
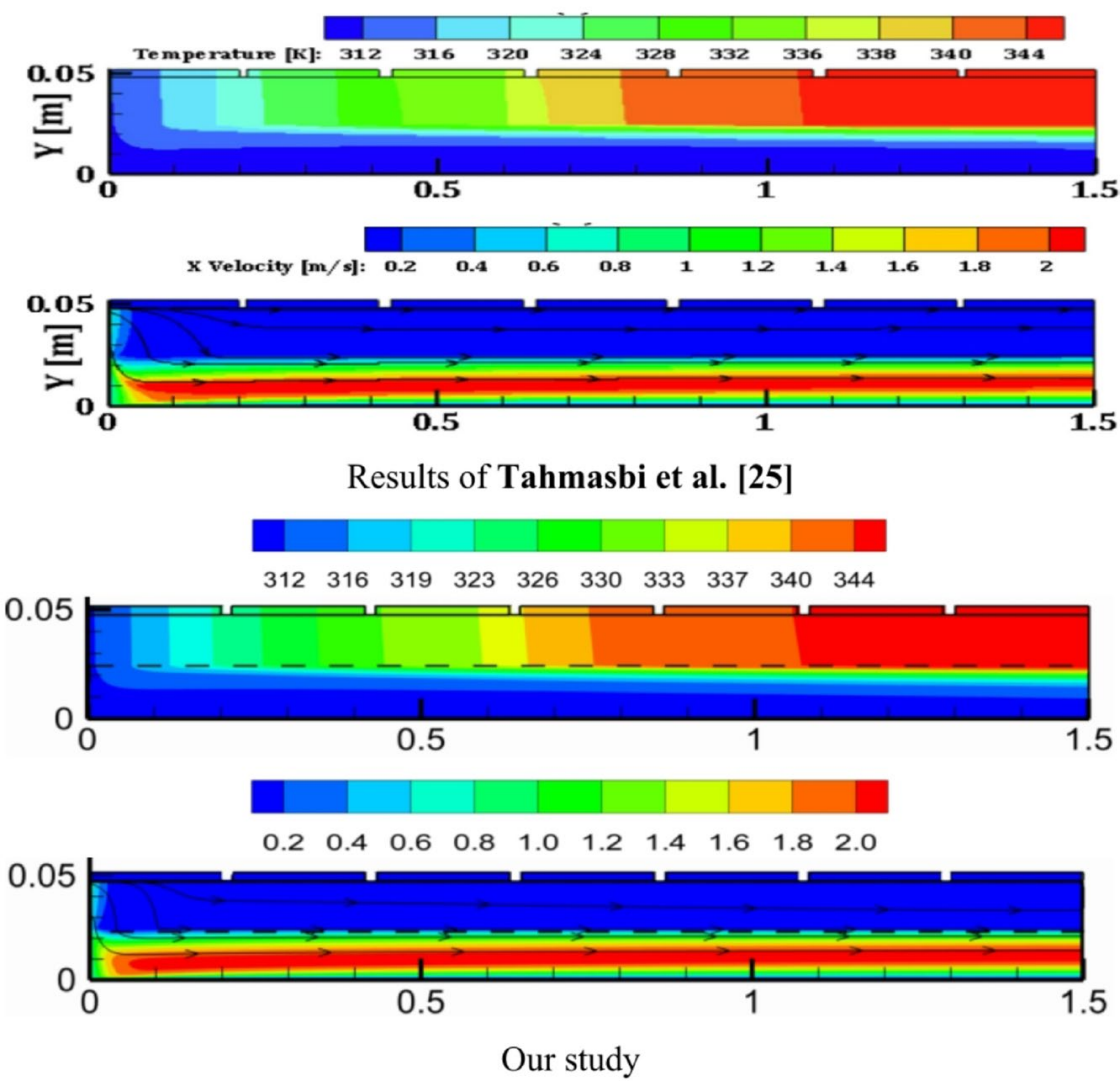
Comparison of dynamic and thermal fields: results of Tahmasbi et al.^25^(on the upper), present study (on the lower) at Ep = H/2, Re = 2300 and, ε = 0.9.

**Fig. 6:**
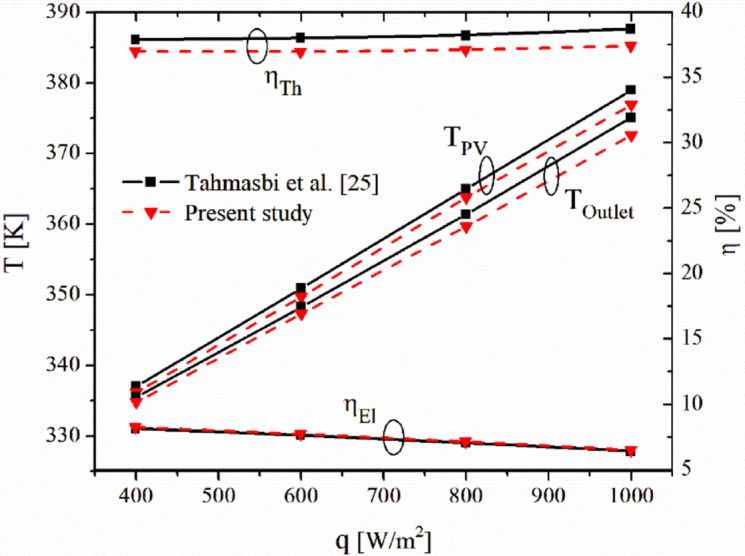
Comparison of the performances of the conventional PVT system generated by our model with those of Tahmasbi et al.^25^.

The second validation was carried out by comparing the present numerical results with those reported by Tahmasbi et al.^25^ for a PV/T solar collector incorporating porous foam to enhance performance. As illustrated in Fig. 5, the predicted flow dynamics and thermal fields show good agreement with the reference results for Ep = H/2, Re = 2300 and ε = 0.9. Furthermore, the performances of the PVT system presented in Fig. 6, along with the corresponding thermal and electrical efficiencies under different solar radiation levels for Ep = H/2, Re = 500 and ε = 0.9, further confirms the accuracy and reliability of the developed numerical model. On the other hand, in order to quantify the performance of our model, a statistical analysis based on four performance indicators including MPE, MBE, RMSE, and R (as defined in the Appendix), has been performed to validate our results against those of Tahmasbi et al.^25^. The findings are summarized in Table 5.

**Table 5:** Statistical analysis of the model validation against the results of Tahmasebi et al.^25^.

Parameter	$\:MPE\:\left[\%\right]$	$\:MBE\:[K/\%]$	$\:RMSE\:[K/\%]$	$\:R\:[-]$
$\:{T}_{PV}$	− 0.3656	− 1.3250	1.4080	0.9919
$\:{T}_{Outlet}$	− 0.4082	− 1.4750	1.6302	0.9878
$\:{\eta\:}_{th}\:[\mathrm{\%}$]	− 2.8507	− 1.0900	1.0993	0.9720
$\:{\eta\:}_{el}\:[\mathrm{\%}$]	0.0850	1.1579	0.0857	0.9825

As presented in Fig. 6; Table 5, the validation of the present model against the results reported by Tahmasbi et al.^25^ demonstrates a very good agreement for both thermal and electrical performance indicators over the studied range of solar radiation (400–1000 W/m²). For the PV cell temperature, the model slightly underestimates the reference data, with a maximum deviation of about 2.1 K at high irradiance levels and a low RMSE of approximately 1.4 K, indicating high predictive accuracy. Similarly, the outlet fluid temperature shows a close match, with deviations not exceeding 2.5 K and an RMSE of around 1.6 K. In terms of efficiencies, the electrical efficiency is accurately captured, with a maximum absolute difference below 0.1% and a very low MPE (≈ 1–2%), confirming the reliability of the electrical model. The thermal efficiency exhibits slightly larger discrepancies, with deviations up to 1.3%, which can be attributed to differences in heat transfer assumptions. Nevertheless, the overall statistical indicators, including high correlation coefficients (*R* > 0.98), confirm that the developed model is capable of reproducing the trends and magnitudes reported in the literature with satisfactory accuracy.

## Results and discussion

In this section, the impact of introducing a solid layer along the lower wall of the channel on the performance of a photovoltaic/thermal (PV/T) solar collector is examined by systematically varying its thickness (Es) across different characteristic lengths (Ls). Subsequently, the effect of the solid layer is coupled with that of the porous layer, which was previously analyzed independently in our earlier work^30^.

The primary objective of this configuration is to intensify convective heat transfer by accelerating airflow as a result of the reduced effective flow cross-section induced by the insertion of the solid layer. The control parameters considered in this study include geometric variables, namely the solid layer thickness (0.1 ≤ Es ≤ 0.7), the solid layer length (0.25 L ≤ Ls ≤ 0.75 L), and the porous layer thickness (0.1 ≤ Ep ≤ 0.7), as well as the Darcy number (Da = 10^− 6^, 10^− 3^, and 10^− 1^). The numerical simulations are performed at a constant low Reynolds number (Re = 500), under an imposed uniform heat flux (q = 1000 W/m^2^), constant Forchheimer number of F = 1, and with a porous layer porosity of ε = 0.98^43,44^.

### **Effect of the geometrical parameters of the of the solid layer (**solid-layer-only configuration)

**Fig. 7:**
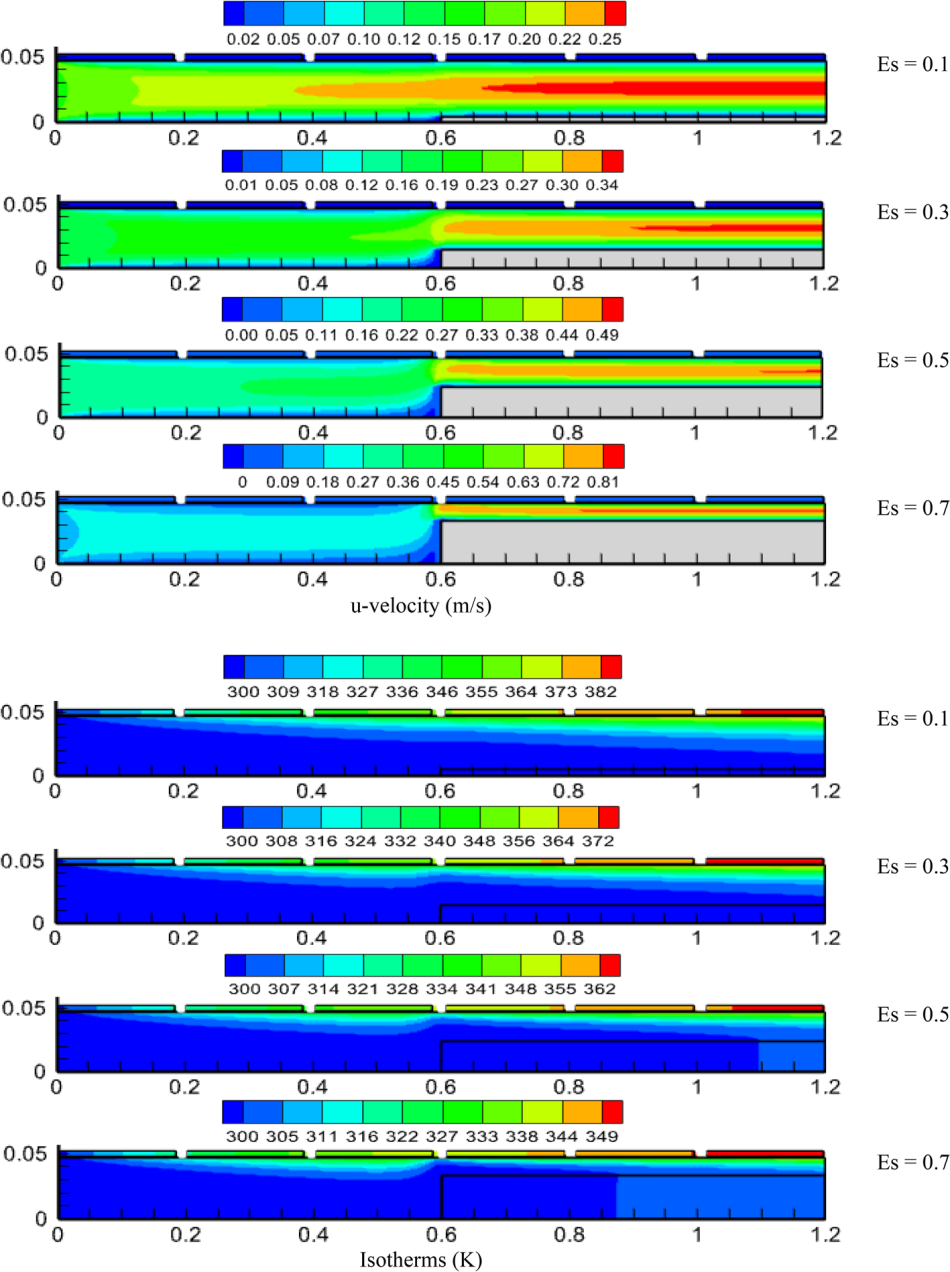
u-velocity contours and isotherms for different values of solid layer thickness Es in the case of without porous layer at Ls = 0.5 L.

**Fig. 8:**
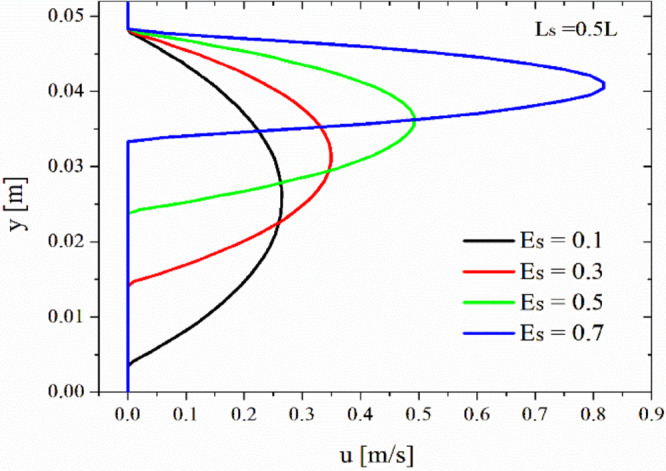
u-velocity profiles as a function of the solid layer thicknesses.

Figure 7 illustrates the effect of increasing the solid-layer thickness on the velocity and temperature fields for a fixed length of Ls = 0.5 L. The velocity contours indicate that a progressive increase in thickness leads to a gradual constriction of the flow passage, resulting in a significant acceleration of the airflow. This trend is further illustrated in Fig. 8, which presents the outlet u-velocity profile of the panel. It can be observed that the maximum velocity increases by approximately 220% as the thickness rises from 0.1 to 0.7. Upstream of the solid layer, the combined effects of a pressure gradient and a local geometric restriction promote the development of recirculation zones, which are reflected by a localized reduction in velocity. The resulting thermal field reveals a pronounced reduction in photovoltaic cell temperature as the solid-layer thickness increases, with the system maximum temperature decreasing by nearly 33 °C. This reduction is mainly attributed to enhanced convective heat transfer, which promotes more effective heat extraction toward the working fluid.

**Fig. 9:**
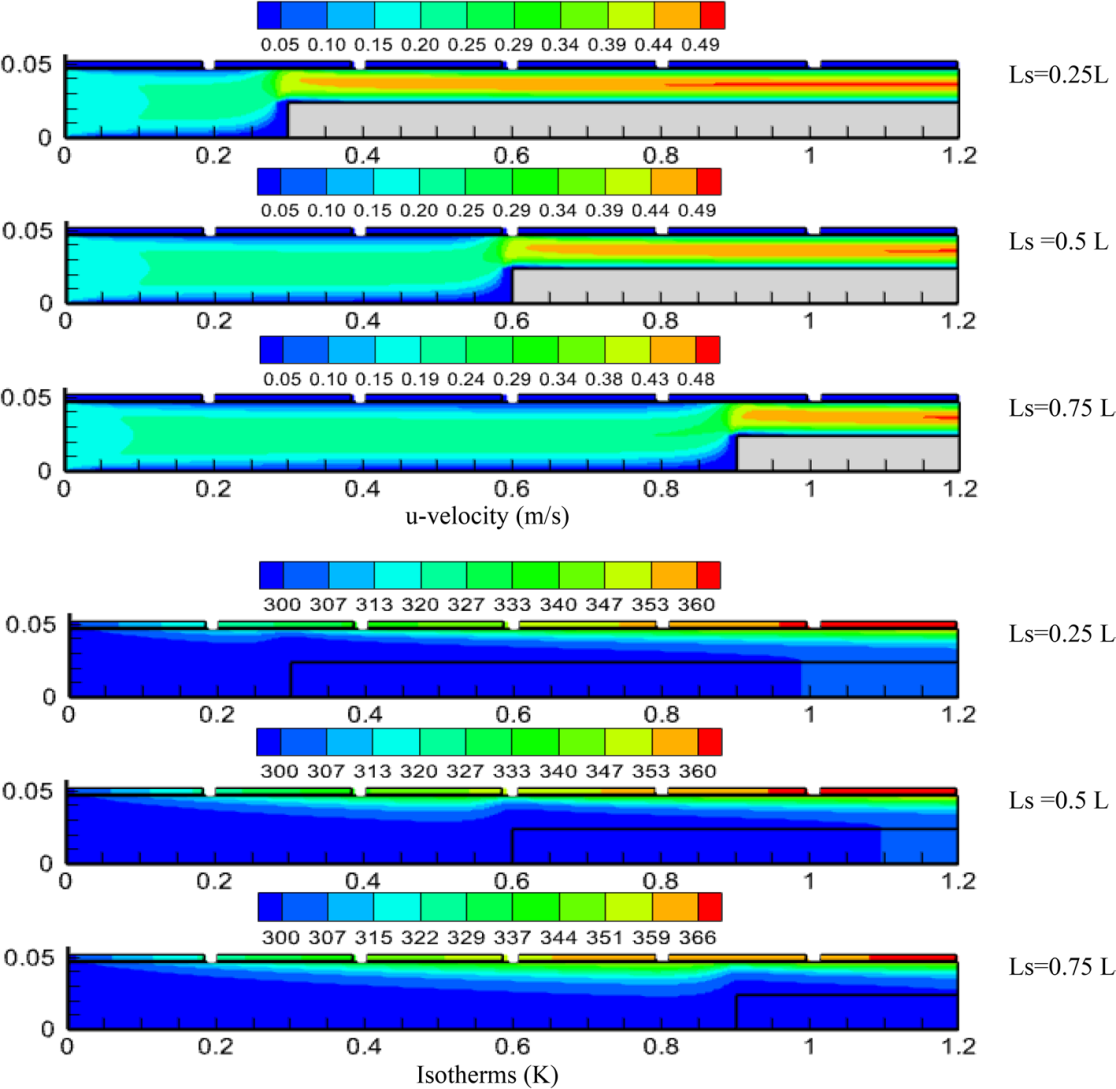
u-velocity contours and isotherms for different values of solid layer length in the case of without porous layer Es = 0.5.

Figure 9 depicts the influence of the solid-layer length on the velocity and thermal fields for a fixed thickness of Es = 0.5. The velocity contours clearly demonstrate that the solid-layer length plays a key role in shaping the velocity field distribution within the channel. When Ls = 0.75, the fluid is rapidly redirected toward the upper region of the channel, producing an extended acceleration zone that persists up to the outlet. Conversely, shortening the solid layer leads to a noticeable reduction in flow velocity in the upstream region over a substantial portion of the channel, depending on the selected value of Ls​. Despite these local variations, the maximum flow velocity remains nearly unchanged across all considered solid-layer lengths. Regarding the thermal fields (isotherms), the maximum temperature of the photovoltaic cells remains nearly constant when the solid-layer length is reduced from Ls = 0.75 L to Ls = 0.5 L. This behavior can be attributed to the fact that, in this configuration, the upstream cells are initially cooler. In contrast, further shortening the layer to Ls = 0.25 L leads to an increase in the maximum cell temperature, reflecting reduced convective heat transfer in the upstream region, where the cells become significantly hotter. This temperature rise is primarily caused by the shortened path of high-velocity airflow over the heated cell surfaces, which limits convective cooling.

**Fig. 10:**
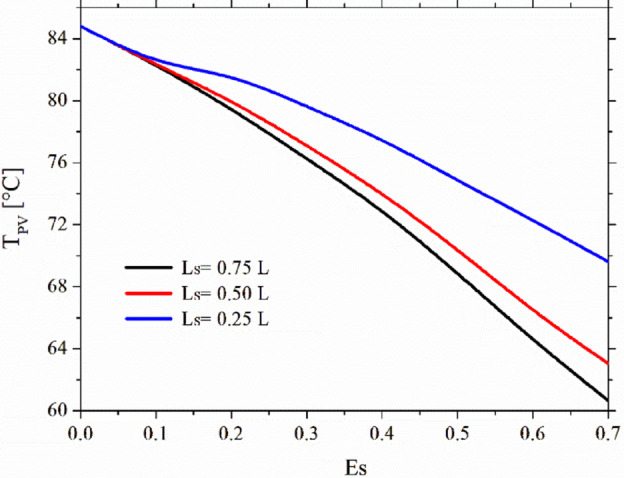
Average PV cells temperature as a function of Es for different values of Ls.

**Fig. 11:**
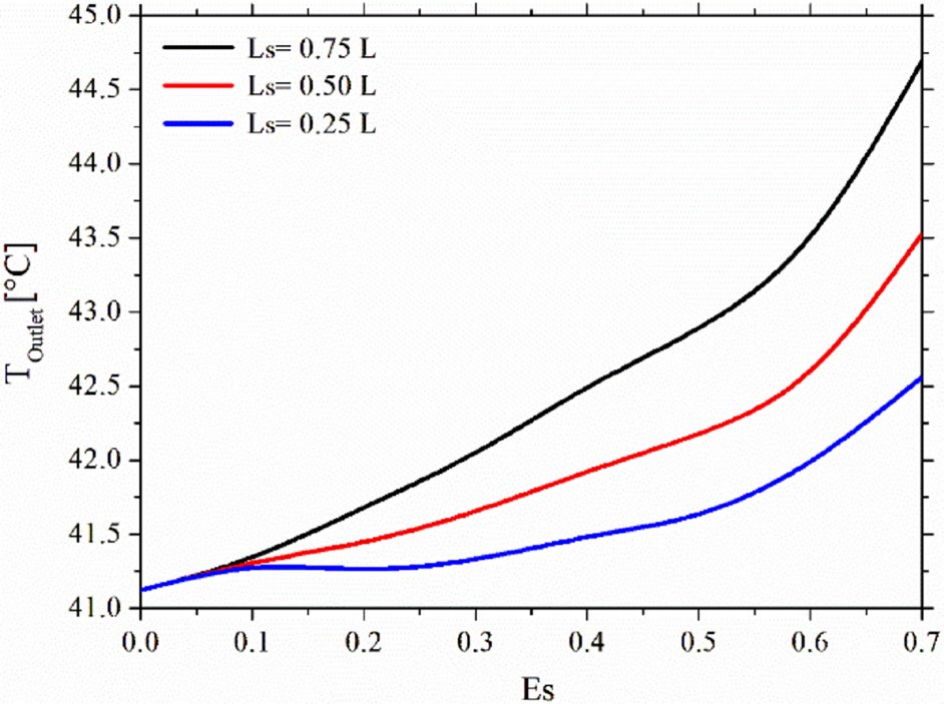
Average air outlet temperature as a function of Es for different values of Ls.

Figures 10 and 11 illustrate the combined effect of the solid-layer thickness (Es​) and length (Ls​) on both the average PV cell temperature (T_PV_, Fig. 10) and the average outlet air temperature (T_Outlet_ ​, Fig. 11). The results indicate that the increasing Es leads to a reduction in the average PV cell temperature primarily due to the acceleration of the airflow in the vicinity of the cells, which enhances convective heat transfer. Consequently, this improvement in heat transfer promotes a rise in the average outlet air temperature. Increasing the solid-layer length (Ls) further enhances the cooling effect, as a larger portion of the PV cells is exposed to high-velocity airflow. The most significant reduction in the average PV cell temperature, reaching 21.67 °C, is obtained for Ls = 0.75 L. Correspondingly, the average outlet air temperature increases by 8.18%, 5.18%, and 3.02% for Ls = 0.75 L, 0.5 L, and 0.25 L, respectively.

**Fig. 12:**
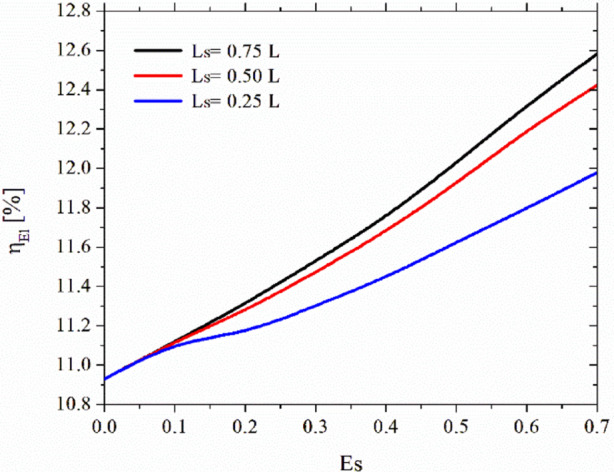
Electrical efficiency as a function of Es for different values of Ls.

**Fig. 13:**
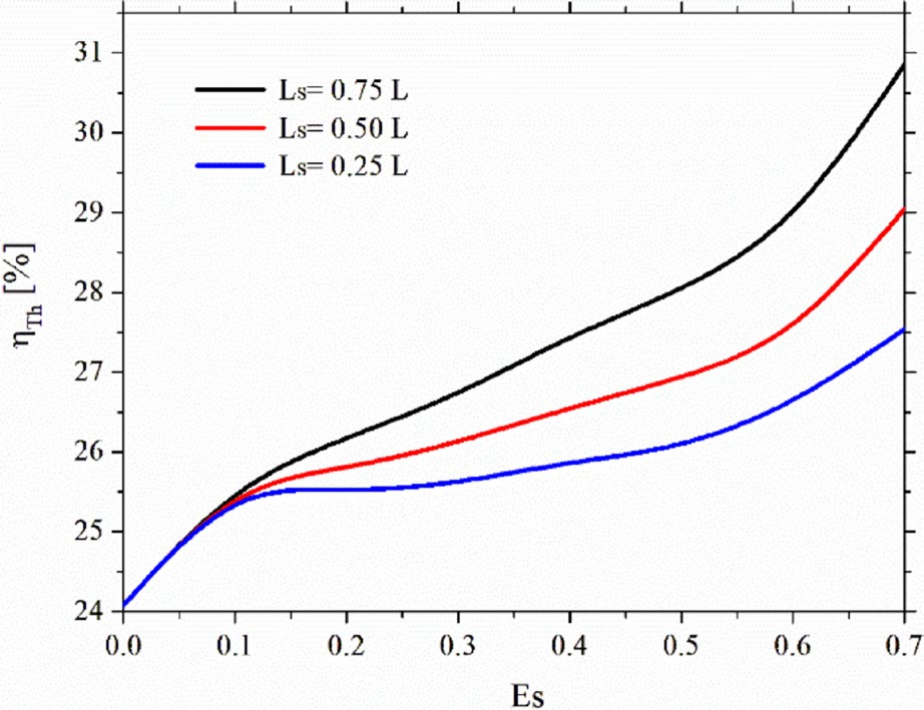
Thermal efficiency as a function of Es for different values of Ls.

Figures 12 and 13 depict respectively the variation of the electrical and thermal efficiencies of the PV/T system with respect to the solid-layer thickness (Es​) and length (Ls​). In agreement with the observations from these figures, increasing both parameters enhance the cooling of the photovoltaic cells and facilitates more effective heat transfer to the working fluid. Consequently, the electrical and thermal efficiencies of the system rise markedly with increasing Es​ and Ls​, attaining maximum values of 12.58% and 30.85%, respectively.

### Combined effect of solid and porous layers

The combined effect of integrating a porous medium with a solid layer on the thermal and electrical performance of the photovoltaic/thermal (PV/T) system is investigated. Particular attention is given to the effects of the solid-layer geometric parameters as well as the Darcy number. It should be noted that, when varying the thickness of the solid layer, the thickness of the porous layer is adjusted simultaneously in order to maintain a constant interlayer spacing of 0.2 between the two layers. Accordingly, as Es increases from 0.1 to 0.7, Ep​ decreases correspondingly from 0.7 to 0.1.

**Fig. 14:**
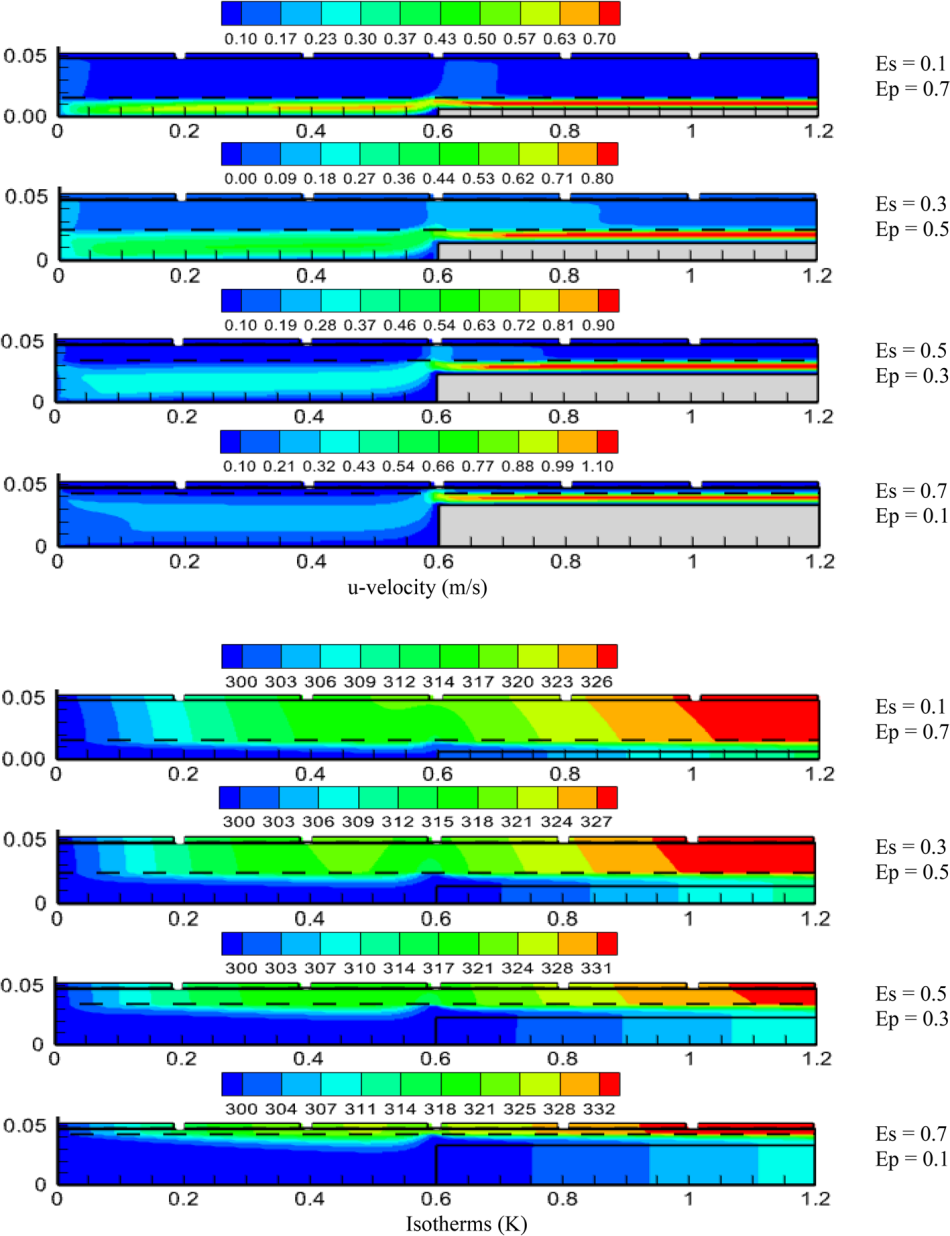
u-velocity contours and isotherms for different values of solid layer thickness Es in the case of with porous layer at Ls = 0.5 L and Da = 10^− 3^.

**Fig. 15:**
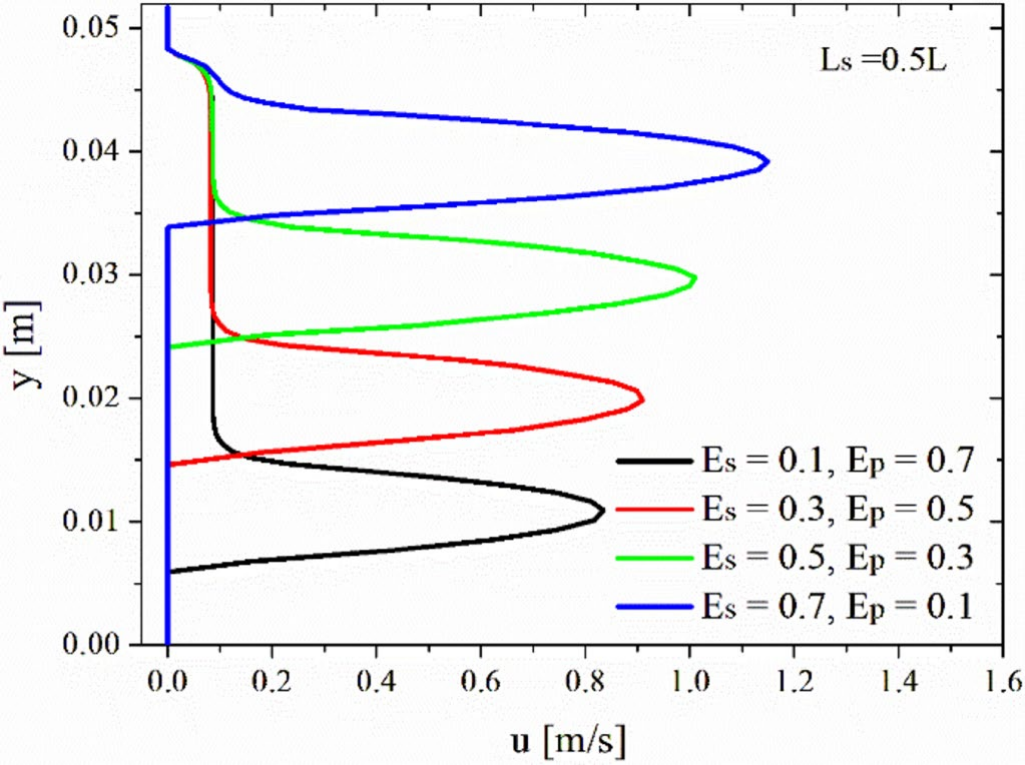
u-velocity profiles as a function of solid and porous layer thicknesses.

Figure 14 illustrates the combined effect of simultaneously increasing the solid-layer thickness (Es​) and decreasing the porous-layer thickness (Ep​) on the flow velocity contours and thermal field for intermediate values of the Darcy number and solid-layer length (Da = 10^− 3^ and Ls = 0.5 L). The dynamic field reveals the presence of a thin, high-velocity air gap above the solid layer. Within the porous layer, the airflow velocity remains relatively low due to the flow resistance imposed by the porous medium. As Es increases from 0.1 to 0.7 (while Ep decreases correspondingly from 0.1 to 0.7), the thin air gap shifts upward toward the PV cells and is accompanied by a noticeable acceleration of the airflow. This behaviour is attributed to the progressive narrowing of the flow passage, resulting in an increase in velocity of approximately 57%. These trends—namely, the upward shift of the air gap and the acceleration of the airflow—are further illustrated by the variation of the u-velocity profile shown in Fig. 15. Upstream of the solid layer, the dynamic field exhibits a marked deceleration of the airflow. In terms of the thermal field, despite the fact that increasing Es enhances convective heat transfer by accelerating the flow, the simultaneous reduction in the porous-layer thickness (Ep) significantly diminishes both conductive heat transfer and the available heat-exchange surface area. Consequently, the maximum PV cell temperature rises by approximately 6 °C.

**Fig. 16:**
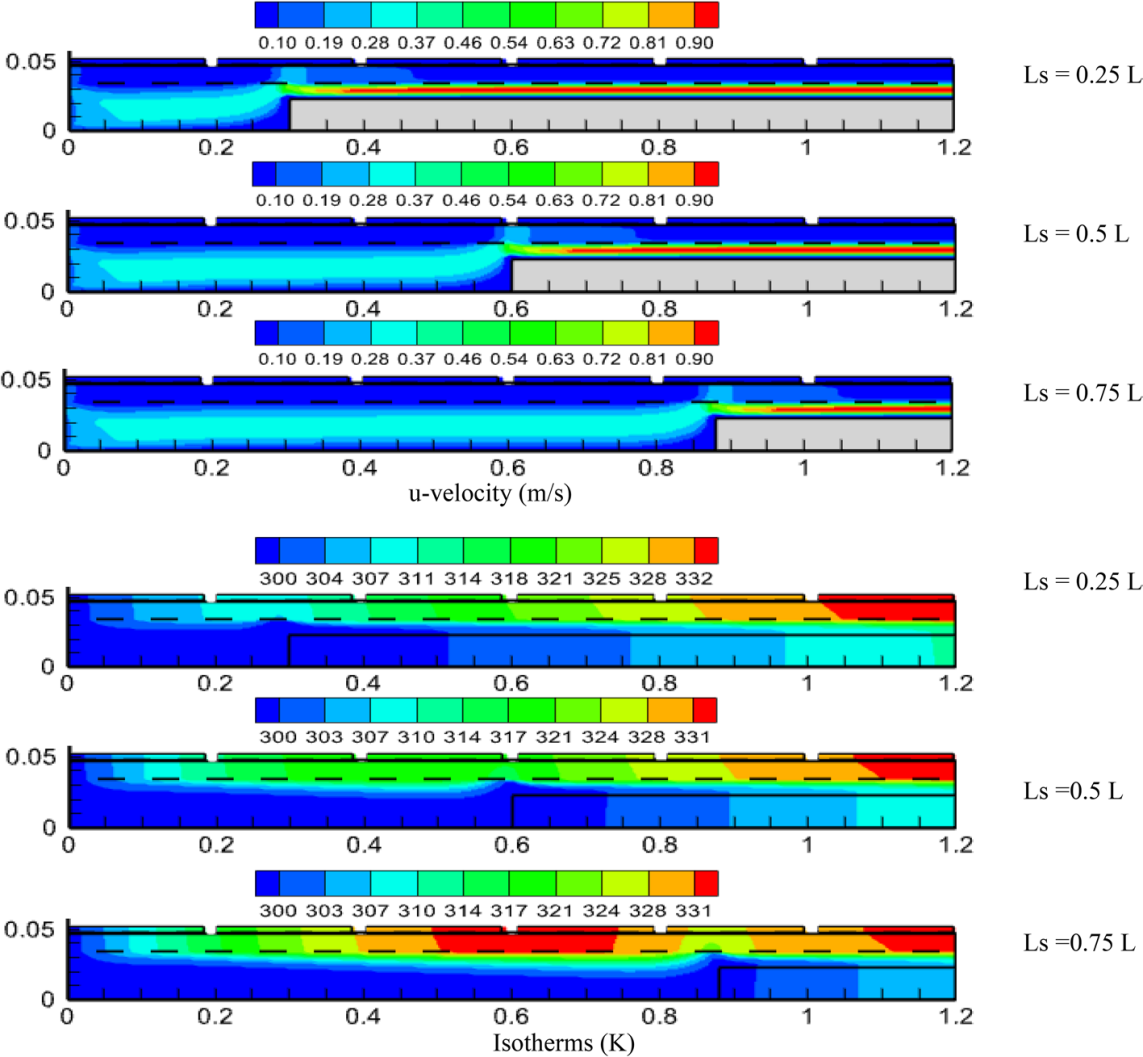
u-velocity contours and isotherms for different values of solid layer length Ls in the case of with porous layer at Ep = 0.3, Es = 0.5 and Da = 10^− 3^.

Figure 16 examines the effect of the solid-layer length on the flow and thermal fields for a fixed solid-layer thickness of 0.5 and a porous-layer thickness of 0.3. The results show that reducing the solid-layer length does not affect the maximum flow velocity in the narrowed section between the two layers, which remains approximately constant at 0.9 m/s. Upstream of the solid layer, the airflow is characterized by relatively low velocities, with a maximum value of about 0.37 m/s. Regarding the thermal field, the maximum temperature of the photovoltaic cells remains nearly unaffected by the variation in the solid-layer length. However, a localized temperature rise is observed upstream of the solid layer, particularly for the shortest length (Ls = 0.25 L). This localized increase can be attributed to a reduction in convective cooling efficiency due to less effective fluid circulation in this region.

**Fig. 17:**
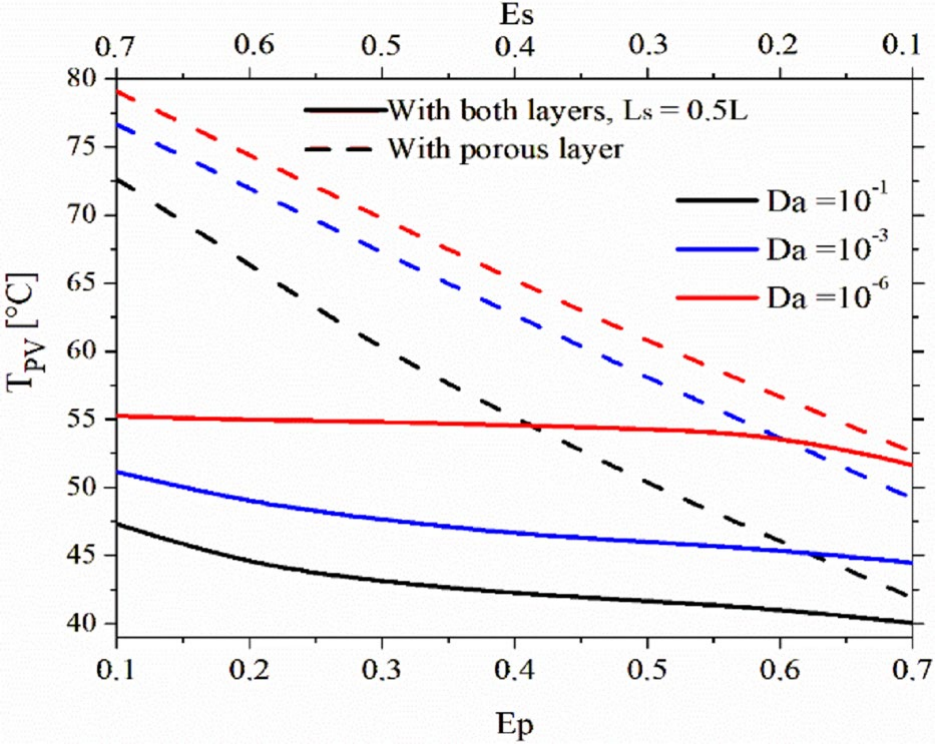
Average PV cells temperature as a function of Ep or Es for different values of Da.

**Fig. 18:**
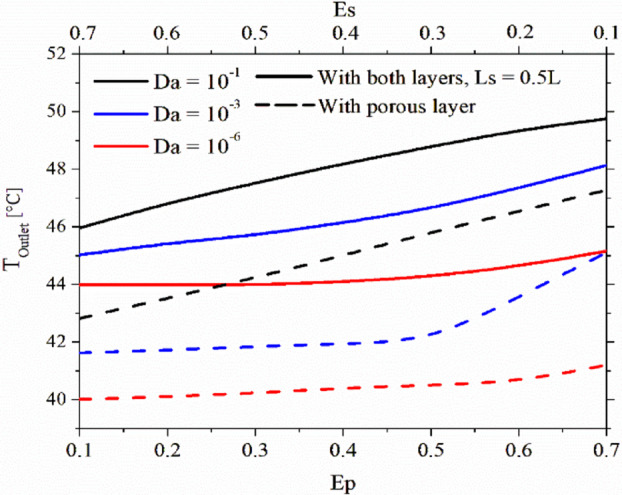
Average air outlet temperature as a function of Ep or Es for different values of Da.

Figures 17 and 18 depict the influence of the governing parameters (Es​, Ep​, and Da) on the average PV cell temperature (Fig. 17) and the average outlet air temperature (Fig. 18), in comparison with the configuration consisting solely of a porous layer. The results confirm earlier findings regarding the effect of the Darcy number (Da), showing that an increase in Da (from 10⁻⁶ to 10^− 1^) leads to a reduction in the PV cell temperature, while simultaneously enhancing the outlet air temperature. In the absence of a solid layer, increasing the porous-layer thickness (Ep​) exerts a more pronounced effect on both temperatures. Moreover, the combined use of a porous layer and a solid layer produces a markedly stronger impact, further reducing the PV cell temperature and elevating the average outlet fluid temperature. This effect is especially significant for large solid-layer thicknesses and thin porous-layer thicknesses (Es = 0.7 and Ep = 0.1), where the average PV cell temperature decreases by approximately 26 °C, while the outlet fluid temperature rises by about 3 °C, compared with the configuration using only the porous layer.

**Fig. 19:**
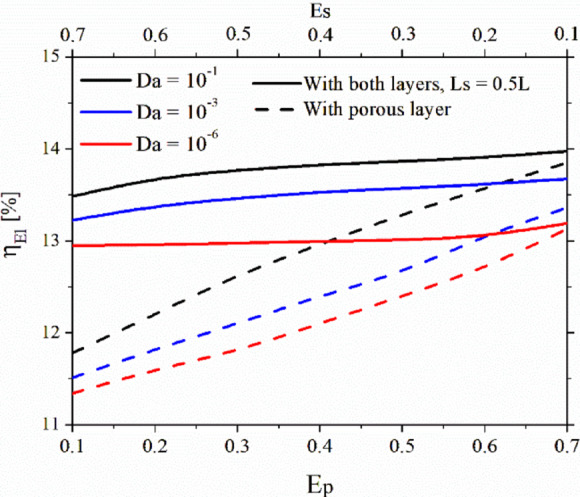
Electrical efficiency as a function of Ep and Es for different values of Da.

**Fig. 20:**
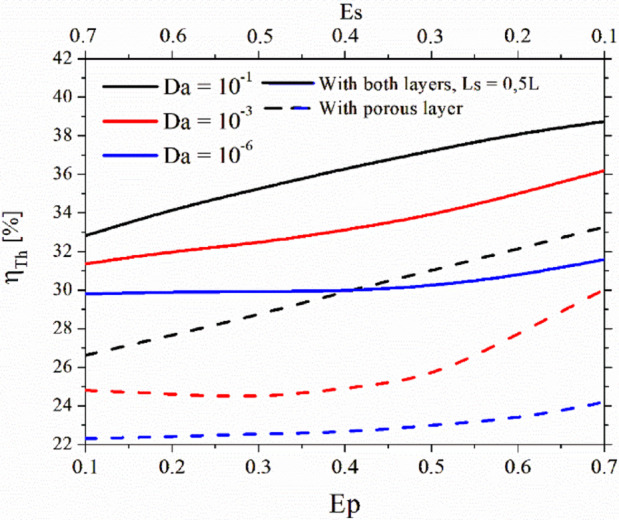
Thermal efficiency as a function of Ep and Es for different values of Da.

The analysis of the electrical and thermal efficiencies presented in Figs. 19 and 20 confirms the preceding observations and further demonstrates that the PV/T system achieves optimal performance when a sufficiently thick porous layer is combined with a highly permeable porous layer (i.e., Ep = 0.7, Da = 10^− 1^). This coupled configuration significantly enhances the cooling of the photovoltaic cells while promoting more efficient heat transfer to the working fluid.

**Fig. 21:**
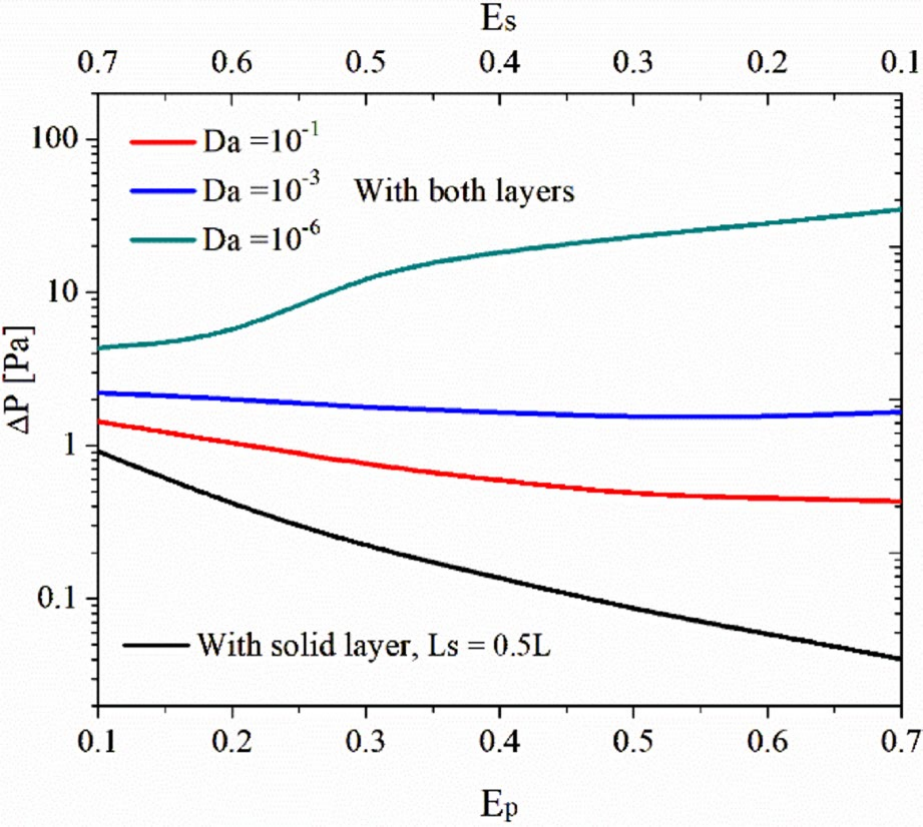
Pressure drop as a function of Ep and Es for different values of Da.

Figure 21 illustrates the influence of both the layer configuration thickness and the Darcy number on the pressure drop and pumping power (presented on a logarithmic scale) at Ls = 0.5 L. For the solid-layer-only configuration, the pressure drop increases markedly with increasing solid layer thickness due to the progressive reduction of the flow passage, resulting in higher resistance. In contrast, the combined solid–porous configuration exhibits a higher pressure drop owing to the additional resistance introduced by the porous medium. For this configuration, two distinct trends are observed depending on the Darcy number. At low Darcy numbers (e.g., Da = 10^− 6^), the porous medium behaves as a highly resistive region, resulting in relatively high pressure drops, which increase with increasing Ep​ (and decreasing Es​). Conversely, at higher Darcy numbers (e.g., Da = 10^− 3^ to 10^− 1^), the permeability is significantly enhanced, facilitating fluid penetration through the porous structure and leading to a reduction in pressure drop. In this regime, the pressure drop exhibits a slight decreasing trend with increasing Ep​ (and decreasing Es​).

**Fig. 22:**
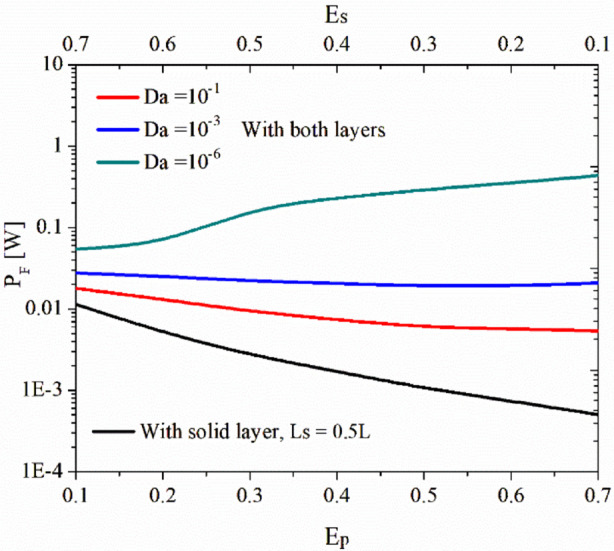
Power fan as a function of Ep and Es for different values of Da.

The variation of pumping power with layer configuration thickness (Ep​ and Es​) for different values of Darcy number is illustrated in Fig. 22. As indicated by Eq. (17), the pumping power is directly proportional to the pressure drop, resulting in similar trends for both quantities. The results show that the pumping power remains relatively low in absolute terms, which can be attributed to the moderate airflow rates considered in this study (Re = 500). However, for the combined solid–porous configuration, P_F_ is significantly higher compared to solid-layer-only configuration, due to the additional flow resistance introduced by the porous medium. This effect becomes more pronounced as the Darcy number decreases and as the porous layer thickness increases (i.e., increasing Ep and decreasing Es​), ultimately reaching its maximum value (P_F_ = 0.435 W) for the most resistive configuration (i.e., Da = 10^− 6^, Ep = 0.7, and Es = 0.1).

For comparative purposes, the optimal electrical and thermal efficiencies of the different configurations, along with the corresponding pressure drop, are summarized in Table 6 for three values of Darcy numbers (Da = 10⁻¹, 10⁻^3^ and 10⁻⁶) at the same Reynolds number (Re = 500). Compared to the conventional configuration (Ep = Es = 0), the solid-layer-only configuration leads to moderate but consistent improvements of approximately 13.6% in electrical efficiency and 20.6% in thermal efficiency. In contrast, the porous-layer-only configuration exhibits a strong dependence on permeability. The electrical efficiency increases by about 20.8% to 25.3%, while the thermal efficiency enhancement ranges from 37.5% at Da = 10⁻¹ to only 0.5% at Da = 10⁻⁶, indicating a drastic reduction in thermal benefit at low permeability due to increased flow resistance. The combined solid–porous configuration delivers the best overall performance. For the configuration (Ep = 0.1, Es = 0.7), the electrical efficiency improves by about 18.0% to 23.3%, while the thermal efficiency increases by approximately 23.5% to 36.2% as the Darcy number varies from 10⁻⁶ to 10⁻¹. Even greater enhancements are obtained for the case of Ep = 0.7, Es = 0.1, where the electrical efficiency rises by up to 27.8%, and the thermal efficiency reaches a maximum improvement of about 60.8% at Da = 10⁻¹. However, these performance gains are accompanied by an increase in pressure drop in configurations involving porous media. As the Darcy number decreases, ΔP rises sharply, reaching high values of 34.8 Pa at Da = 10⁻⁶, highlighting the pronounced hydraulic penalty at low permeability.

Overall, although configurations with a thick porous layer provide the highest thermal and electrical enhancements, a combination of a relatively thin solid layer with a thick porous layer offers a more balanced and practical compromise, ensuring satisfactory performance while limiting pressure losses and reducing material cost, particularly at intermediate Darcy numbers (Da ≥ 10⁻³).

**Table 6:** Maximum thermal and electrical efficiencies for studied configurations.

	Without both layers	With solid layer	With porous layer			With both porous and solid layer					
	Ep = Es=0	Es = 0.7	Ep = 0.7			Ep = 0.1, Es = 0.7			Ep = 0.7, Es = 0.1		
Da	–	–	10^− 1^	10^− 3^	10^− 6^	10^− 1^	10^− 3^	10^− 6^	10^− 1^	10^− 3^	10^− 6^
$\:{\eta\:}_{el}$	10.93	12.42	13.7	13.38	13.2	13.48	13.22	12.86	13.97	13.67	13.19
$\:{\eta\:}_{th}$	24.08	29.04	33.1	29.81	24.2	32.81	31.36	29.74	38.73	36.2	31.58
$\:\varDelta\:P\:$	0.032	0.915	0.258	0.753	31.69	1.432	2.21	4.35	0.432	1.66	34.8

## Conclusion

In this study, the combined integration of a solid layer and a porous medium within a photovoltaic/thermal (PV/T) system was numerically investigated to evaluate its impact on thermal and electrical performance. The influence of key parameters—including solid-layer thickness and length, the porous-layer thickness, and the Darcy number—on system performance was systematically evaluated. The obtained results demonstrate that:

- The incorporation of a solid layer significantly enhances convective heat transfer by accelerating the airflow in the vicinity of the PV cells, resulting in a 3.75 °C increase in outlet fluid temperature and a 24.4 °C reduction in the average PV cell temperature. Consequently, the system achieves improvements of 20.6% and 13.6% in thermal and electrical efficiencies, respectively, compared with the baseline configuration (Es = Ep = 0).
- Comparative analysis reveals that the use of a porous layer alone with high permeability (Da = 10⁻¹) significantly enhances system performance compared with a solid layer configuration, yielding increases of up to 37.45% and 25.35% in thermal and electrical efficiencies, respectively. Nevertheless, the most effective thermo-hydraulic performance is achieved through the combined configuration of a thin solid layer and a thick porous layer (Es = 0.1 and Ep = 0.7). Under this optimal arrangement, the PV/T system exhibits remarkable improvements of approximately 60% in thermal efficiency and 28% in electrical efficiency relative to the baseline case, while keeping pressure drop within acceptable limits.
- The hydraulic performance of the PV/T system is strongly influenced by both the layer configuration and the Darcy number. At low Darcy numbers (Da ≤ 10⁻³), the combined solid–porous configuration results in a significantly higher pressure drop due to the strong flow resistance imposed by the porous medium.
- Regardless of the investigated configurations, the pumping power remains relatively low in absolute terms, which can be attributed to the moderate airflow conditions considered in this study (Re = 500), resulting in limited system energy consumption.
- From a cost-efficiency perspective, the optimal design corresponds to a configuration featuring a thick solid layer coupled with a thin porous medium, which reduces material costs while maintaining satisfactory thermal and electrical performance.

Finally, a further extension of this work is strongly recommended to investigate the validity of the local thermal non-equilibrium (LTNE) assumption within the porous layer. Under this framework, the solid matrix and the fluid phase are allowed to exhibit distinct local temperatures, leading to a more realistic representation of heat transfer phenomena. In such a case, additional parameters must be considered, including the interphase heat transfer coefficient and the effective thermal conductivity ratio.

In parallel, a comprehensive techno-economic assessment of the proposed PVT system will be conducted to complement the present thermal analysis. This will involve the development of detailed cost models accounting for material properties, manufacturing processes, and system integration costs, as well as the evaluation of key economic indicators such as capital investment, operation and maintenance costs, and levelized cost of energy. Furthermore, a multi-objective optimization framework will be implemented to simultaneously evaluate thermal performance and economic feasibility, enabling the identification of an optimal design based on combined thermo-economic criteria. Such an extension will provide a more rigorous basis for design recommendations and enhance the practical applicability of the proposed system.

## Data Availability

The datasets used and/or analysed during the current study available from the corresponding author on reasonable request.

## Appendix

The validation of the developed model to quantify the performances of the developed model against those of the literature is performed based on four statistical parameters namely Means Bias Error (MBE), Mean Percentage Error (MPE), Root Mean Square Error (RMSE), and Coefficient of correlation (R), which are written as follows:

18 $$\:MBE=\:\frac{1}{n}\sum\:_{i=1}^{n}\left({x}_{predicted}-{x}_{literature}\right)$$

19 $$\:MPE=\:\frac{100\%}{n}\sum\:_{i=1}^{n}\left(\frac{{x}_{literature}-{x}_{predicted}}{{x}_{literature}}\right)$$

20 $$\:RMSE=\:\sqrt{\sum\:_{i=1}^{n}\frac{{({x}_{predicted}-{x}_{literature})}^{2}}{n}}$$

21 $$\:R=\:\frac{n\:\left(\sum\:{x}_{literature}\:\times\:{x}_{predicted}\right)-(\sum\:{x}_{literature})\times\:(\sum\:{x}_{predicted})}{\sqrt{\left[n\sum\:{x}_{literature}^{2}-{(\sum\:{x}_{literature})}^{2}\right]\times\:\left[\:n\sum\:{x}_{predicted}^{2}-{(\sum\:{x}_{predicted})}^{2}\right]}}$$

Where: $\:n$, $\:{x}_{literature}$, $\:{x}_{predicted}$ are the number of data points, the value from literature (Tahmasbi et al.^25^, and estimated one respectively.

## Abbreviations

A_c_: PV cell area (m^2^)
C_p_: Specific heat capacity (J kg^−1^ K^−1^)
${C}_{f}$: Coefficient of form drag
Da: Darcy number
Ep: Dimensionless thickness of the porous layer
Es: Dimensionless thickness of the solid layer
F: Forchheimer number
Fp: Fan power (W)
g: Gravitational acceleration (m s^−2^)
H: Channel height (m)
h_p_: Porous layer thickness (m)
h_pv_: Height of the PV cell (m)
I: Solar irradiation (W m^−2^)
K: Permeability (m^2^)
$$k$$: Thermal conductivity (W m^−1^ K^−1^)
L: Length of the panel (m)
L_S_: Length of the solid layer (m)
L_p_: Length of the porous layer (m)
L_PV_: Length of the PV cell (m)
$\dot{m}$: Mass flow rate (kg s^−1^)
MBE: Means Bias Error (K/%)
MPE: Mean Percentage Error (%)
p: Pressure (Pa)
ΔP: Pressure drop (Pa)
P: Dimensionless pressure
Pr: Prandtl number
Q_u_: Useful thermal energy (W)
R: Coefficient of correlation
Re: Reynolds number
RMSE: Root Mean Square Error (K/%)
Rµ: Dynamic viscosity ratio
Kr: Thermal conductivity ratio
T: Temperature (K)
u: x-velocity component (m s^−1^)
U: Dimensionless x-velocity component
v: y-velocity component (m s^−1^)
V: Dimensionless y-velocity component
U_int_: Air inlet velocity (m s^−1^)
x, y: Cartesian coordinates (m)
X, Y: Dimensionless Cartesian coordinates

## Subscripts

El: Electrical
eff: Effective
f: Fluid
Fan: Fan
int: Inlet
Motor: Motor
Outlet: Outlet
p: Porous
PV: PV cell
ref: Reference
s: Solid
th: Thermal

## Greek symbols

${\beta}_{0}$: Silicon efficiency temperature coefficient (K^−1^)
θ: Dimensionless temperature
ε: Porosity
ρ: Density (kg m^−3^)
ƞ: Efficiency (%)
μ: Dynamic viscosity (Pa s)
$\uplambda$: Coefficient used in the momentum equation
